# Supplementary material for: A dynamic partitioning mechanism polarizes membrane protein distribution
Source: Nat Commun. 2023 Nov 30;14:7909. doi: 10.1038/s41467-023-43615-2 (PMC10689845; doi:10.1038/s41467-023-43615-2)
Supplement: Supplementary file 1 — Supplementary Information [file 41467_2023_43615_MOESM1_ESM.pdf]

A dynamic partitioning mechanism polarizes membrane protein distribution

Tatsat Banerjee[1,2,*], Satomi Matsuoka[3,4], Debojyoti Biswas[5], Yuchuan Miao[1,6], Dhiman Sankar Pal[1], Yoichiro Kamimura[3], Masahiro Ueda[3,4], Peter N. Devreotes[1,6,*], Pablo A. Iglesias[1,5,*].

[1]Department of Cell Biology and Center for Cell Dynamics, School of Medicine, Johns Hopkins University, Baltimore, MD, USA.

[2]Department of Chemical and Biomolecular Engineering, Whiting School of Engineering, Johns Hopkins University, Baltimore, MD, USA.

[3]Laboratory for Cell Signaling Dynamics, RIKEN Center for Biosystems Dynamics Research, Suita, Osaka, Japan.

[4]Laboratory of Single Molecule Biology, Graduate School of Frontier Biosciences, Osaka University, Suita, Osaka, Japan.

[5]Department of Electrical and Computer Engineering, Whiting School of Engineering, Johns Hopkins University, Baltimore, MD, USA.

[6]Department of Biological Chemistry, School of Medicine, Johns Hopkins University, Baltimore, MD, USA.

* Corresponding authors. Email: tatsatb@jhu.edu (to T.B.), pnd@jhmi.edu (to P.N.D.), pi@jhu.edu (to P.A.I.).

SUPPLEMENTARY INFORMATION

This file contains:

- Supplementary Figures 1-14.
- Supplementary Tables 1-4.

Other supplementary materials include:

- Videos S1-S24.
- Description of Additional Supplementary Files.

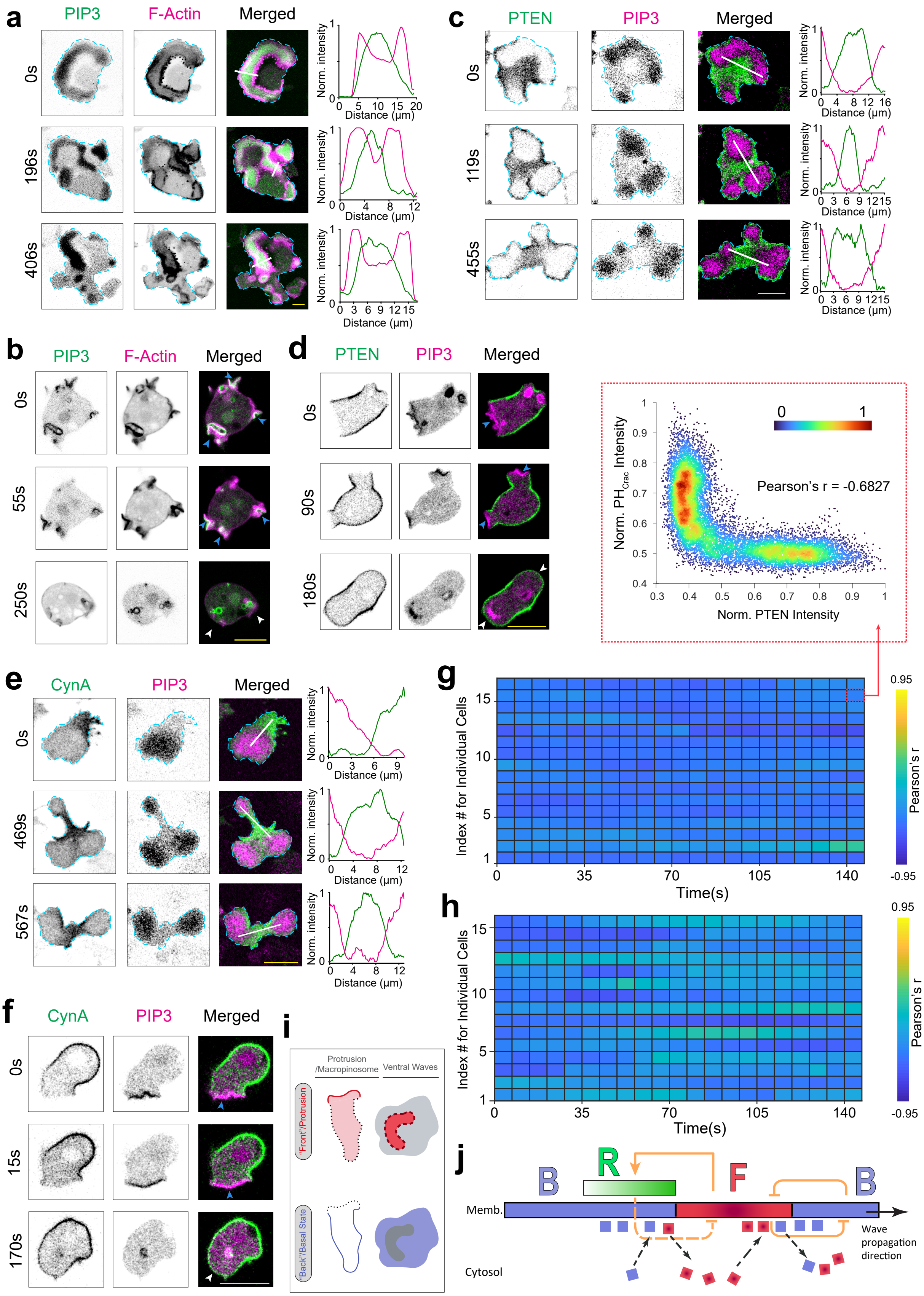

a
PIP3
F-Actin
Merged
0s
196s
406s
Norm. intensity
Distance (μm)
c
PTEN
PIP3
Merged
0s
119s
455s
b
PIP3
F-Actin
Merged
0s
55s
250s
d
PTEN
PIP3
Merged
0s
90s
180s
Norm. PH$_{Crac}$ Intensity
Norm. PTEN Intensity
Pearson's r = -0.6827
e
CynA
PIP3
Merged
0s
469s
567s
g
Index # for Individual Cells
Time(s)
Pearson's r
0.95
-0.95
h
Index # for Individual Cells
Time(s)
Pearson's r
0.95
-0.95
f
CynA
PIP3
Merged
0s
15s
170s
i
Protrusion
/Macropinosome
Ventral Waves
"Front"/Protrusion
"Back"/Basal State
j
R
B
F
B
Memb.
Cytosol
Wave
propagation
direction

SUPPLEMENTARY FIGURE 1 | Formation of two mutually exclusive dynamic states on the plasma membrane.

(a) Representative live-cell images of cortical waves in the substrate attached ventral surface of a *Dictyostelium* cell co-expressing PI(3,4,5)P3 biosensor PH_{Crac}-GFP and newly-polymerized actin biosensor $LimE_{\Delta coil}$-mCherry. Line-scan intensity profiles are shown in the rightmost panels. Unless otherwise mentioned, throughout the study, line-scan intensity profiles are shown in rightmost or bottommost panels; times are always indicated in seconds in left or top.

(b) Representative live-cell images of protrusion formation in migrating *Dictyostelium* cell co-expressing PH_{Crac}-GFP and $LimE_{\Delta coil}$-mCherry, demonstrating their co-localization on protrusions.

(c-f) Representative live-cell images of ventral wave propagation (c, e) and protrusion formation (d, f) in *Dictyostelium* cells expressing either PTEN-GFP (c, d) or CynA-KikGR (e, f), along with PH_{Crac}-mCherry. In (b), (d), and (f), blue arrowheads: Protrusions enriched in PH_{Crac} and $LimE_{\Delta coil}$; white arrowheads: the membrane domains that returned to the back/basal state.

(g, h) Heatmap demonstrating the consistent complementarity of PTEN (g) and CynA (h) with respect to PIP3. Throughout the study, the extent of complementary or co-localization was quantified in terms of Pearson's correlation coefficient (r) with respect to PIP3 and n_f=20 frames were analyzed (7s/frame) for each cell. Heatmaps were plotted in "Parula" colormap. For PTEN, number of cell, n_c=16 (g); for CynA, n_c=15 (h). (g) inset shows the red channel vs green channel normalized intensity correlation plot of one frame for one cell (in "Turbo" colormap).

(i) Schematic showing the front-back complementarity in migrating cell protrusions (1D profiles on left) and and cortical waves on ventral surface (2D profiles on right). In either situation, whenever a front-state is created from the back- or basal- state of the membrane, back markers were faithfully depleted from that particular domain.

(j) F-B-R (Front-Back-Refractory) excitable topology showing positive and negative feedbacks inside biochemical networks and relative spatiotemporal position of F, B, and R molecules in membrane. F molecules are shown as red squares and B molecules are shown as blue squares. As "B"-state on the membrane were being converted to "F"-state, B molecules dissociated from membrane and went to cytosol, whereas, F molecules were recruited to the membrane. The exact opposite sequence of events happened when an "F"-state was reverting to B-state. For all figures in this study, scale bars are 10 μm. Source data are provided as a Source Data file.

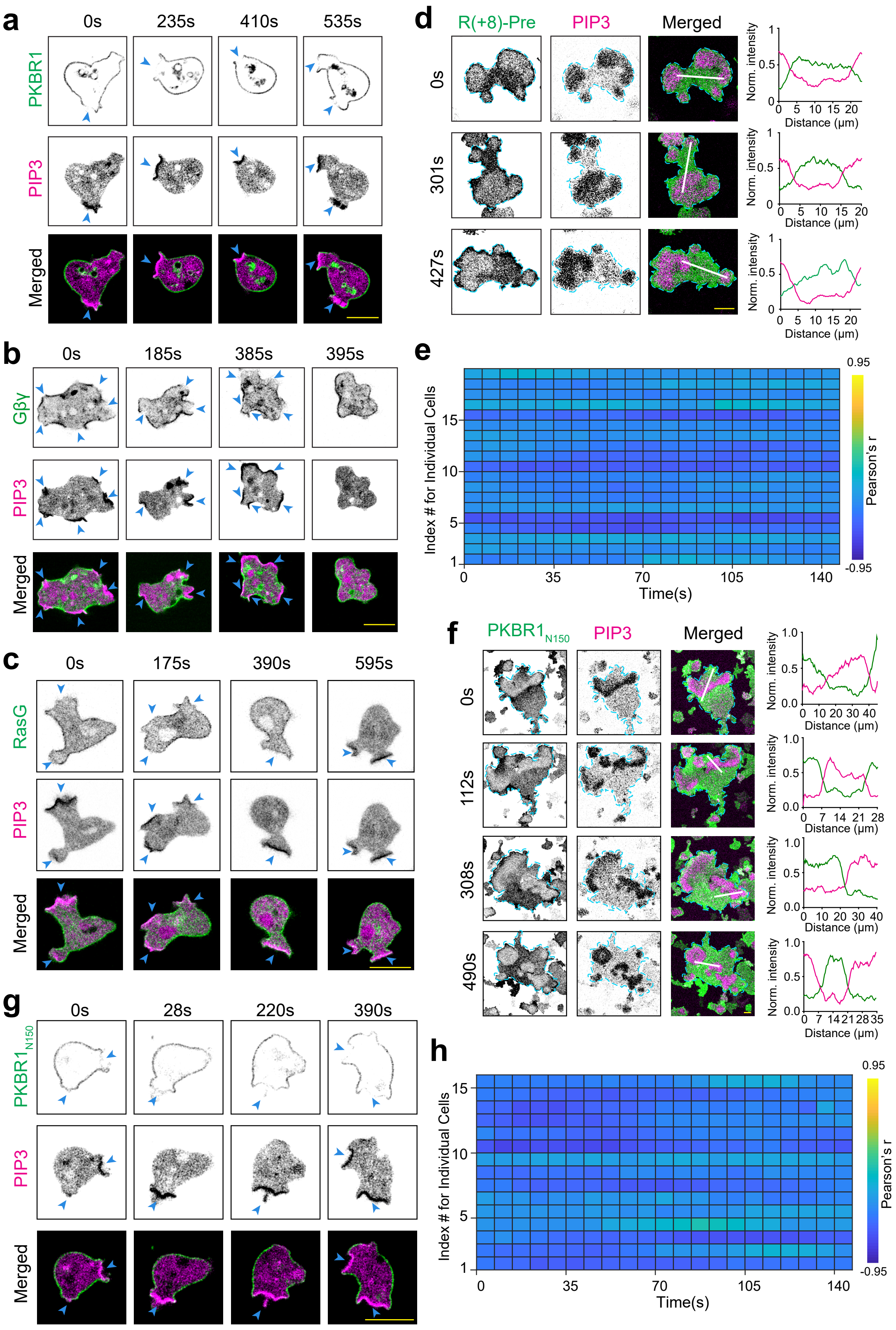

a
0s
235s
410s
535s
PKBR1
PIP3
Merged
b
0s
185s
385s
395s
Gβγ
PIP3
Merged
c
0s
175s
390s
595s
RasG
PIP3
Merged
d
R(+8)-Pre
PIP3
Merged
0s
301s
427s
Norm. intensity
Distance (μm)
e
Index # for Individual Cells
Time(s)
Pearson's r
0.95
-0.95
f
PKBR1N150
PIP3
Merged
0s
112s
308s
490s
Norm. intensity
Distance (μm)
g
0s
28s
220s
390s
PKBR1N150
PIP3
Merged
h
Index # for Individual Cells
Time(s)
Pearson's r
0.95
-0.95

Supplementary Figure 2 | **Dynamic distribution of different endogenous and synthetic lipidated membrane proteins, with respect to PIP3 levels, during ventral wave propagation and amoeboid migration.**

(a-c) Representative live-cell images of protrusion formation in migrating *Dictyostelium* cell co-expressing PH_{Crac}-mCherry along with PKBR1-KikGR(a), or KikGR-Gβ (b), or GFP-RasG (c), showing PKBR1, G$\beta\gamma$, and RasG were consistently depleted from protrusions. Blue arrowheads: PIP3-rich protrusions.
(d) Representative live-cell images of ventral wave propagation in *Dictyostelium* cell co-expressing PH_{Crac}-mCherry and surface charge sensor GFP-R(+8)-Pre showing persistent depletion of surface charge sensor from the activated-regions of the membrane.
(e) Pearson's r heatmap showing extent of complementary localization between PIP3 and R(+8)-Pre over time for different individual cells. n_c=19 cells.
(f, g) Representative live-cell images of ventral wave propagation (f) and protrusion formation (g) in *Dictyostelium* cells co-expressing PH_{Crac}-mCherry and $PKBR1_{N150}$-GFP, demonstrating consistent complementary localization of $PKBR1_{N150}$ and PIP3 over membrane. In (g), blue arrowheads: PIP3-rich protrusions.
(h) Pearson's r heatmap showing extent of complementary localization between PIP3 and $PKBR1_{N150}$ over time for different individual cells. n_c=15 cells.
Source data are provided as a Source Data file.

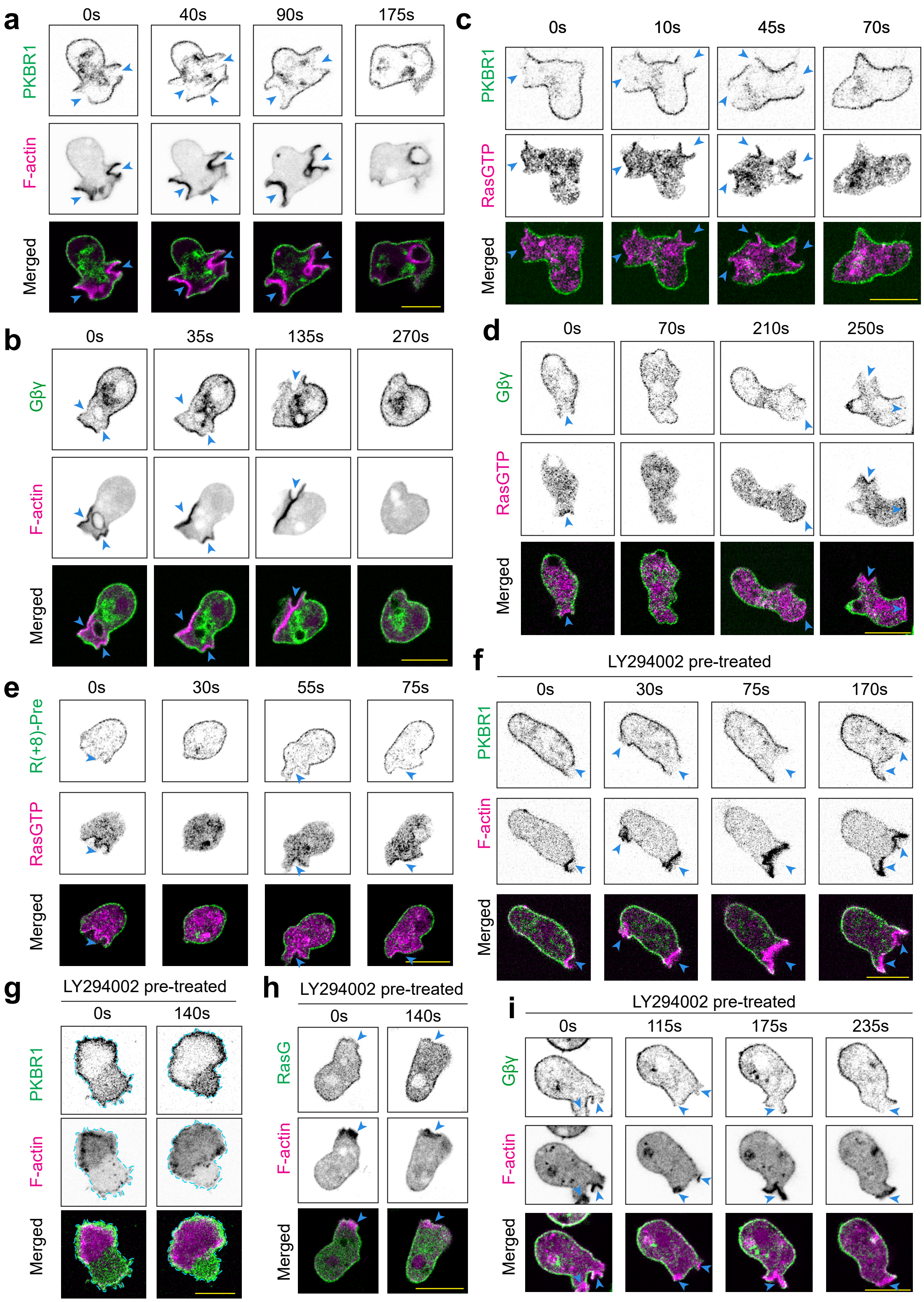
a
0s
40s
90s
175s
PKBR1
F-actin
Merged
b
0s
35s
135s
270s
Gβγ
F-actin
Merged
c
0s
10s
45s
70s
PKBR1
RasGTP
Merged
d
0s
70s
210s
250s
Gβγ
RasGTP
Merged
e
0s
30s
55s
75s
R(+8)-Pre
RasGTP
Merged
f
LY294002 pre-treated
0s
30s
75s
170s
PKBR1
F-actin
Merged
g
LY294002 pre-treated
0s
140s
PKBR1
F-actin
Merged
h
LY294002 pre-treated
0s
140s
RasG
F-actin
Merged
i
LY294002 pre-treated
0s
115s
175s
235s
Gβγ
F-actin
Merged

Supplementary Figure 3 | **Distribution of different endogenous and synthetic lipidated proteins, with respect to biosensors for RasGTP and newly polymerized F-actin, during protrusion formation.**

(a, b) Representative live-cell images of protrusion formation in migrating *Dictyostelium* cell co-expressing LimE-mCherry and PKBR1-KikGR(a) or KikGR-Gβ (b). Blue arrowheads: Protrusions enriched with LimE biosensor.

(c-e) Representative live-cell images of protrusion formation in migrating *Dictyostelium* cell co-expressing RBD-RFP (biosensor for activated Ras) and PKBR1-KikGR (c), KikGR-Gβ (d), or GFP-R(+8)-Pre (e). Blue arrowheads: Protrusions enriched with RBD.

(f, g) Representative live-cell images of protrusion formation (f) and ventral wave propagation (g) in *Dictyostelium* cells co-expressing PKBR1-KikGR and LimE-mCherry. Cells were pre-treated with 40 μM LY294002. Blue arrowheads: Protrusions enriched with LimE.

(h, i) Representative live-cell images of protrusion formation in *Dictyostelium* cells expressing LimE-mCherry, along with GFP-RasG (h) or KikGR-Gβ (i). Cells were pre-treated with 40 μM LY294002. Blue arrowheads: Protrusions enriched with LimE.

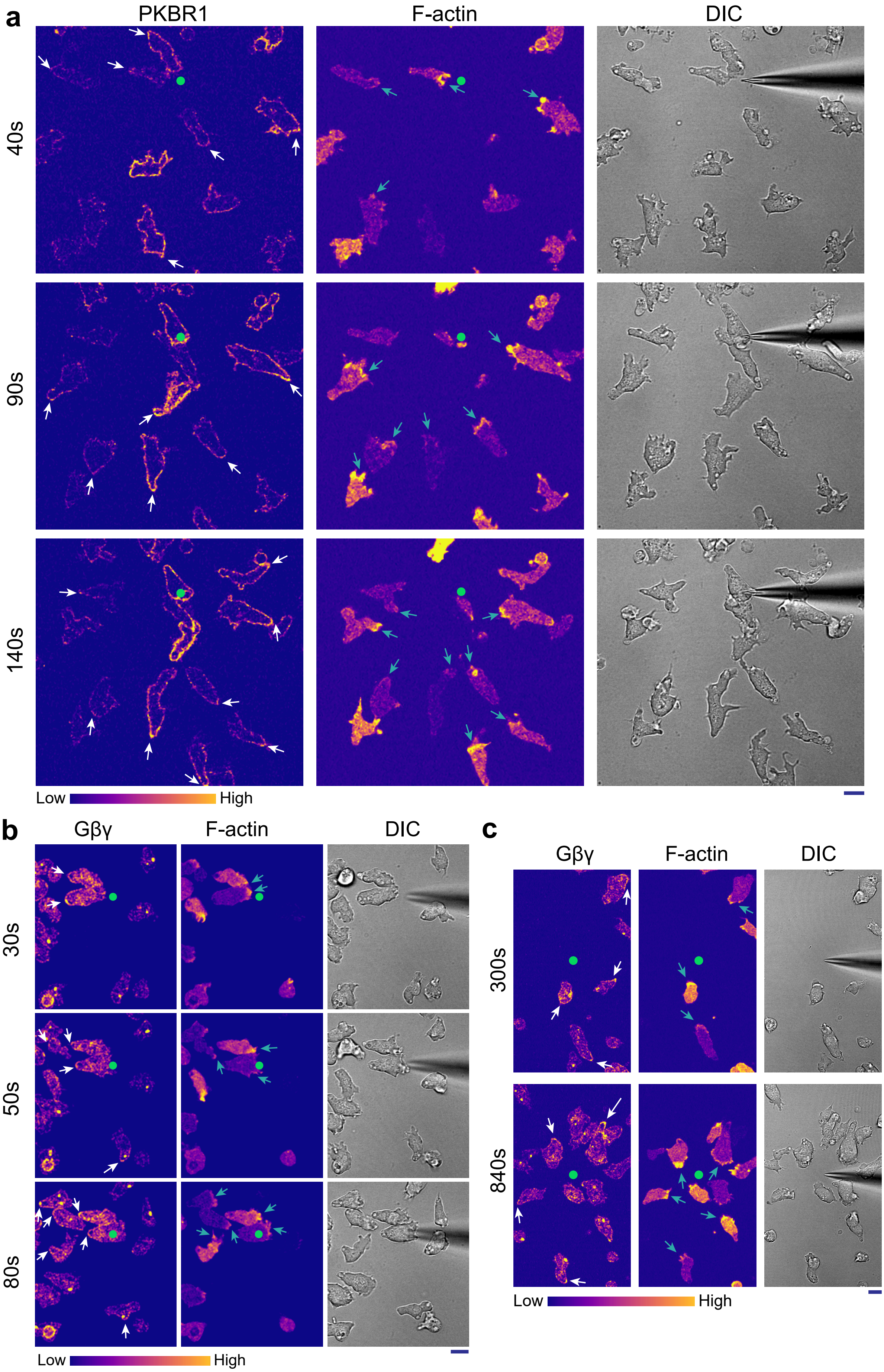
a
PKBR1
F-actin
DIC
40s
90s
140s
Low
High
b
Gβγ
F-actin
DIC
30s
50s
80s
Low
High
c
Gβγ
F-actin
DIC
300s
840s
Low
High

Supplementary Figure 4 | **Localization of lipid-anchored proteins during chemotaxis.**

(a) Representative live-cell images of *Dictyostelium* cell, co-expressing PKBR1-KikGR and LimE-mCherry, chemotaxing towards a 10 μM cAMP-filled micropipette. In (a) to (c), fluorescent images are shown in matplotlib "Plasma" colormap. Green circles in fluorescent channels showing the tip of the micropipette (drawn based on the micropipette shown in the DIC channel). White arrows: PKBR1 enriched in back of the cell membrane, Sea Green arrows: LimE-rich protrusions.

(b, c) Two examples image series of *Dictyostelium* cell, co-expressing KikGR-Gβ and LimE-mCherry, chemotaxing towards a 10 μM cAMP-filled micropipette. White arrows: Gβ enriched in back of the cell membrane, Sea Green arrows: LimE-rich protrusions.

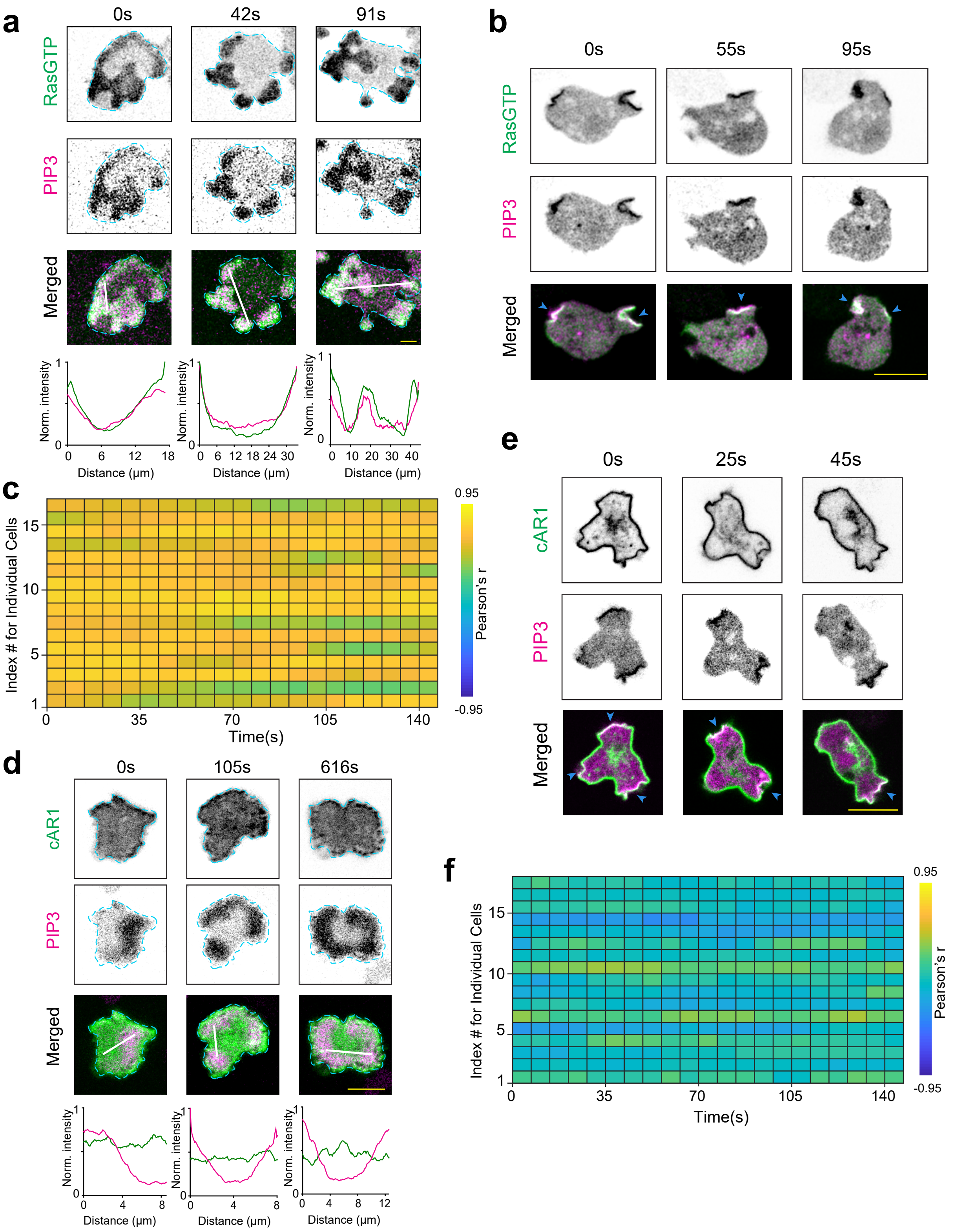

Supplementary Figure 5 | **Spatiotemporal dynamics of Ras activation and surface receptor localization.**

(a, b) Representative live-cell images of ventral wave propagation (a) and protrusion formation (b) in *Dictyostelium* cells co-expressing PH_{Crac}-mCherry and RasGTP biosensor RBD-GFP, demonstrating consistent co-localization of activated Ras and PIP3 in two different physiological scenarios. In (b), blue arrowheads: Protrusions enriched in both PH_{Crac} and RBD.
(c) Pearson's r heatmap showing extent of co-localization between PIP3 and activated Ras over time for different individual cells. n_c=16 cells.
(d, e) Representative live-cell images of ventral wave propagation (d) and protrusion formation (e) in *Dictyostelium* cells co-expressing PH_{Crac}-mCherry and cAR1-GFP, demonstrating consistent uniform distribution of cAR1 across protrusion/front-state and back-state. In (e), blue arrowheads: PIP3-rich Protrusions.
(f) Pearson's r heatmap showing extent of co-/complementary-localization between PIP3 and cAR1 over time for different individual cells. n_c=17 cells.
Source data are provided as a Source Data file.

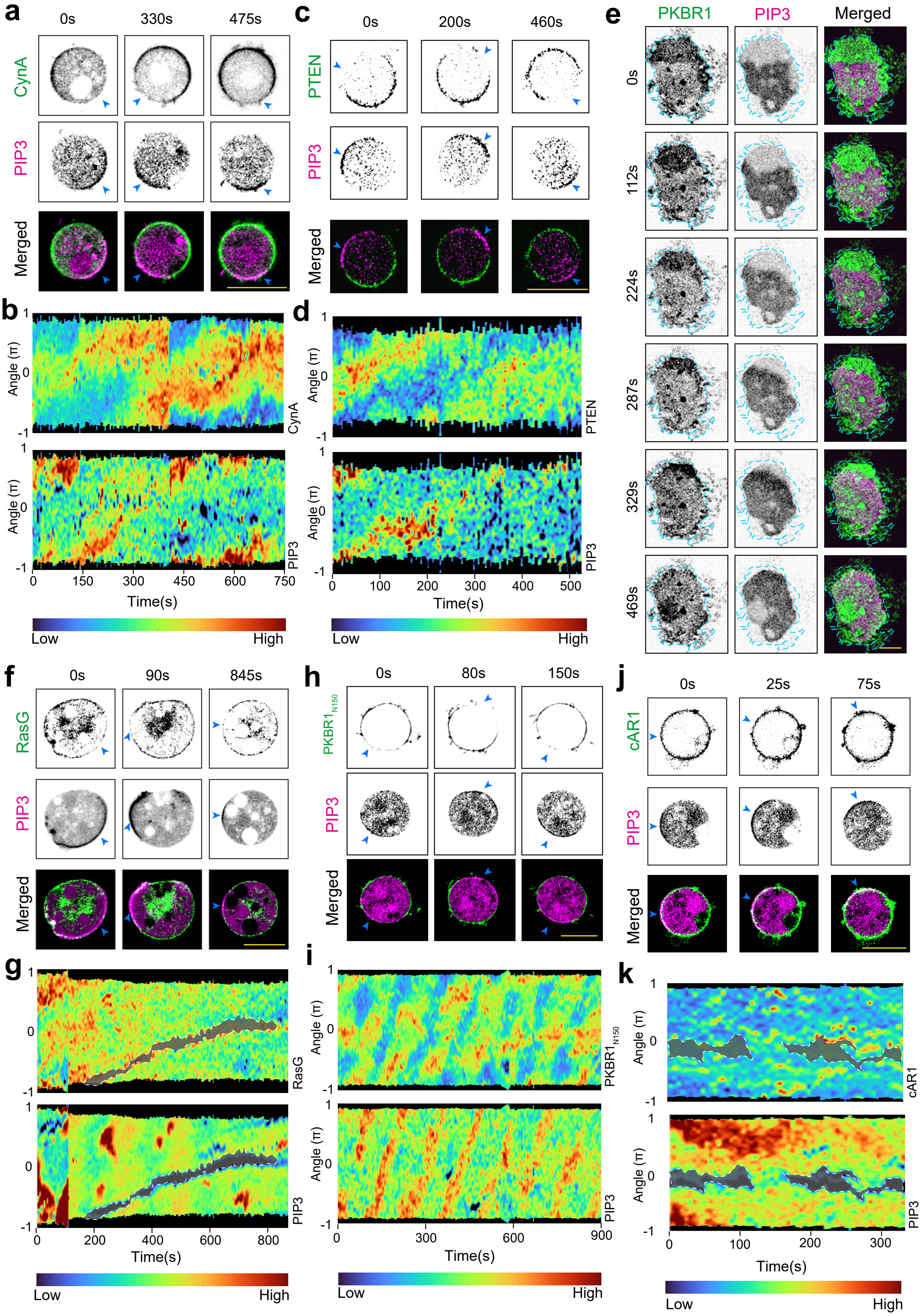
a
0s
330s
475s
CynA
PIP3
Merged
b
Angle (π)
CynA
PIP3
Time(s)
Low
High
c
0s
200s
460s
PTEN
PIP3
Merged
d
Angle (π)
PTEN
PIP3
Time(s)
Low
High
e
PKBR1
PIP3
Merged
0s
112s
224s
287s
329s
469s
f
0s
90s
845s
RasG
PIP3
Merged
g
RasG
PIP3
Time(s)
Low
High
h
0s
80s
150s
PKBR1N150
PIP3
Merged
i
Angle (π)
PKBR1N150
PIP3
Time(s)
Low
High
j
0s
25s
75s
cAR1
PIP3
Merged
k
Angle (π)
cAR1
PIP3
Time(s)
Low
High

Supplementary Figure 6 | Dynamic polarization of multiple back-state associated membrane proteins in cells where actin polymerization was inhibited

(a-d) Representative live-cell time-lapse images (a, c) and 360° membrane kymographs (b, d) of asymmetric 1D wave propagation in *Dictyostelium* cell co-expressing PH_{Crac}-mCherry along with CynA-KikGR (a, b) or PTEN-GFP (c, d) showing consistent depletion of CynA as well as PTEN from the front-state regions of membrane marked by PIP3. In (a-k), cells were pre-treated with actin polymerization inhibitor Latrunculin A (final concentration 5 μM).

(e) Representative live-cell images of *Dictyostelium* cell co-expressing PKBR1-KikGR and PH_{Crac}-mCherry, where the substrate-attached ventral surface was imaged, showing consistent complementarity between PKBR1 and PIP3 during 2D wave propagation, even in the absence of actin cytoskeleton.

(f, g) Representative live-cell images (f) and 360° membrane kymographs (g) of asymmetric 1D wave propagation in *Dictyostelium* cell co-expressing PH_{Crac}-mCherry and GFP-RasG showing near complementary distribution of RasG with respect to front-state regions marked by PIP3.

(h, i) Representative live-cell time-lapse images (h) and 360° membrane kymographs (i) of asymmetric 1D wave propagation in *Dictyostelium* cell co-expressing PH_{Crac}-mCherry and $PKBR1_{N150}$-GFP showing consistent depletion of $PKBR1_{N150}$ from the front-state regions of membrane marked by PIP3.

(j, k) Representative live-cell time-lapse images (j) and 360° membrane kymographs (k) of asymmetric 1D wave propagation in *Dictyostelium* cell co-expressing PH_{Crac}-mCherry and cAR1-GFP showing uniform distribution of cAR1 over the entire membrane. In (a), (c), (f), (h), and (j), blue arrowheads are showing front-state regions of membrane marked by PIP3.

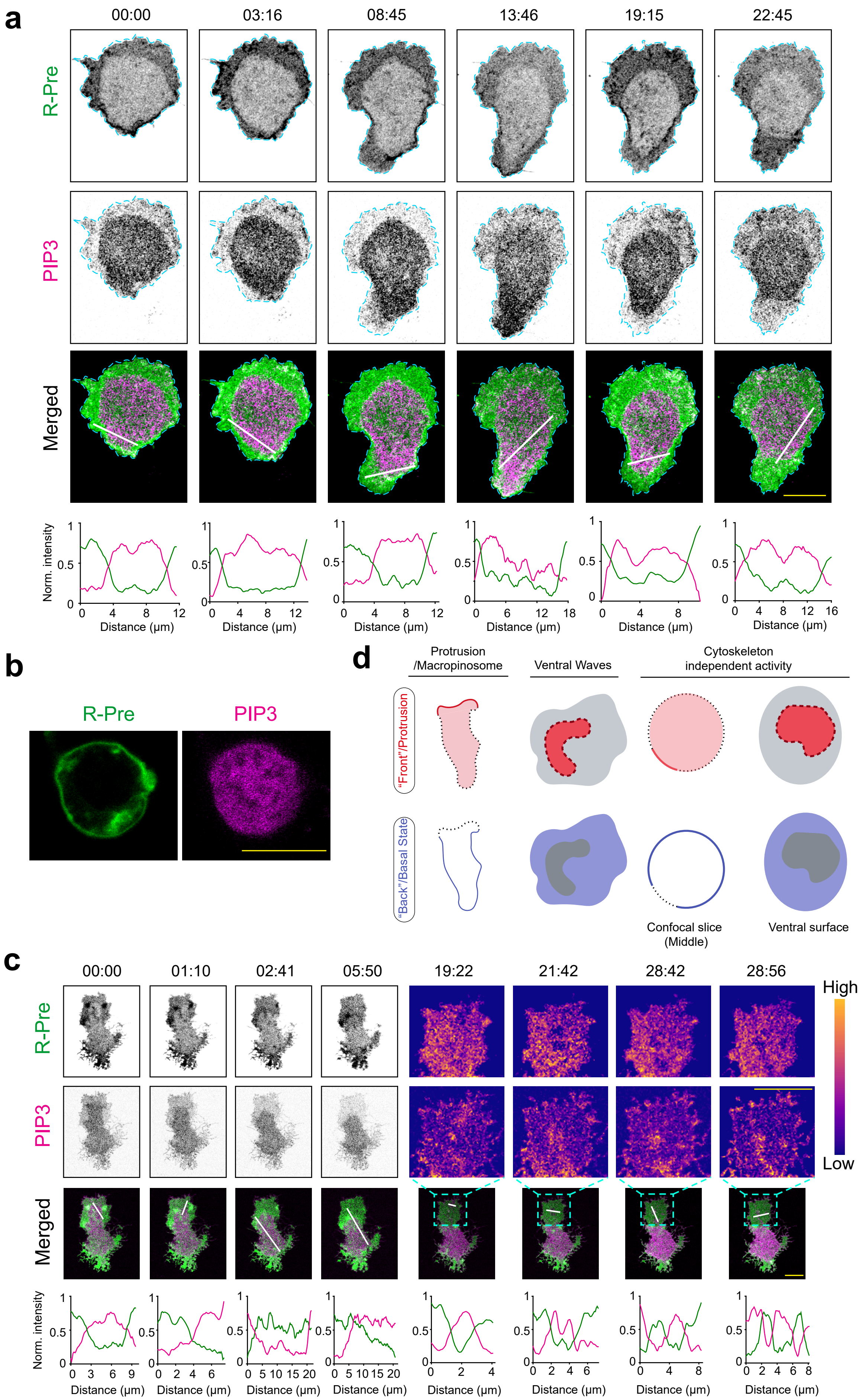
a
00:00
03:16
08:45
13:46
19:15
22:45
R-Pre
PIP3
Merged
Norm. intensity
Distance (µm)
b
R-Pre
PIP3
d
Protrusion /Macropinosome
Ventral Waves
Cytoskeleton independent activity
"Front"/Protrusion
"Back"/Basal State
Confocal slice (Middle)
Ventral surface
c
00:00
01:10
02:41
05:50
19:22
21:42
28:42
28:56
High
Low

Supplementary Figure 7 | **Dynamic asymmetric localization of a lipid-anchored protein in RAW 264.7 macrophages.**

(a) Live-cell images of RAW 264.7 macrophages co-expressing GFP-R(+8)-Pre and PH_{Akt}-mCherry showing ventral wave propagation in the substrate-attached surface of the cell. Time in mm:ss format. Note that, R(+8)-Pre and PH_{Akt}-mCherry are showing consistent complementarity during entire time of the wave propagation.

(b) Live-cell images of RAW 264.7 macrophages co-expressing GFP-R(+8)-Pre and PH_{Akt}-mCherry, where cells were pre-incubated with 5 μM Latrunculin A and 50 μM of ROCK inhibitor Y-27632, before putting them on the opsonized substrate.

(c) Live-cell images of RAW 264.7 macrophages co-expressing GFP-R(+8)-Pre and PH_{Akt}-mCherry showing ventral wave propagation in the substrate-attached surface of the cell. Note that, cell were first pre-incubated with 50 μM of ROCK inhibitor Y-27632, then allowed to spread on the opsonized substrate, and then finally 5 μM Latrunculin A was added (Latrunculin A was added 20 min before starting the image acquisition). The magnified versions of aqua colored rectangular regions in composite images (bottom panels) are shown in matplotlib Plasma colormap (top two panels) in last four time-points. Note that either when big waves were propagating (first four time points) or small wavelets were generated (last four time points), R(+8)-Pre consistently moved away from PIP3-rich regions (also shown in linescan profiles at the bottom). Time in mm:ss format.

(d) Schematic showing that complementary dynamics between front-state and back-state associated membrane molecules are conserved in cytoskeleton impaired cells.

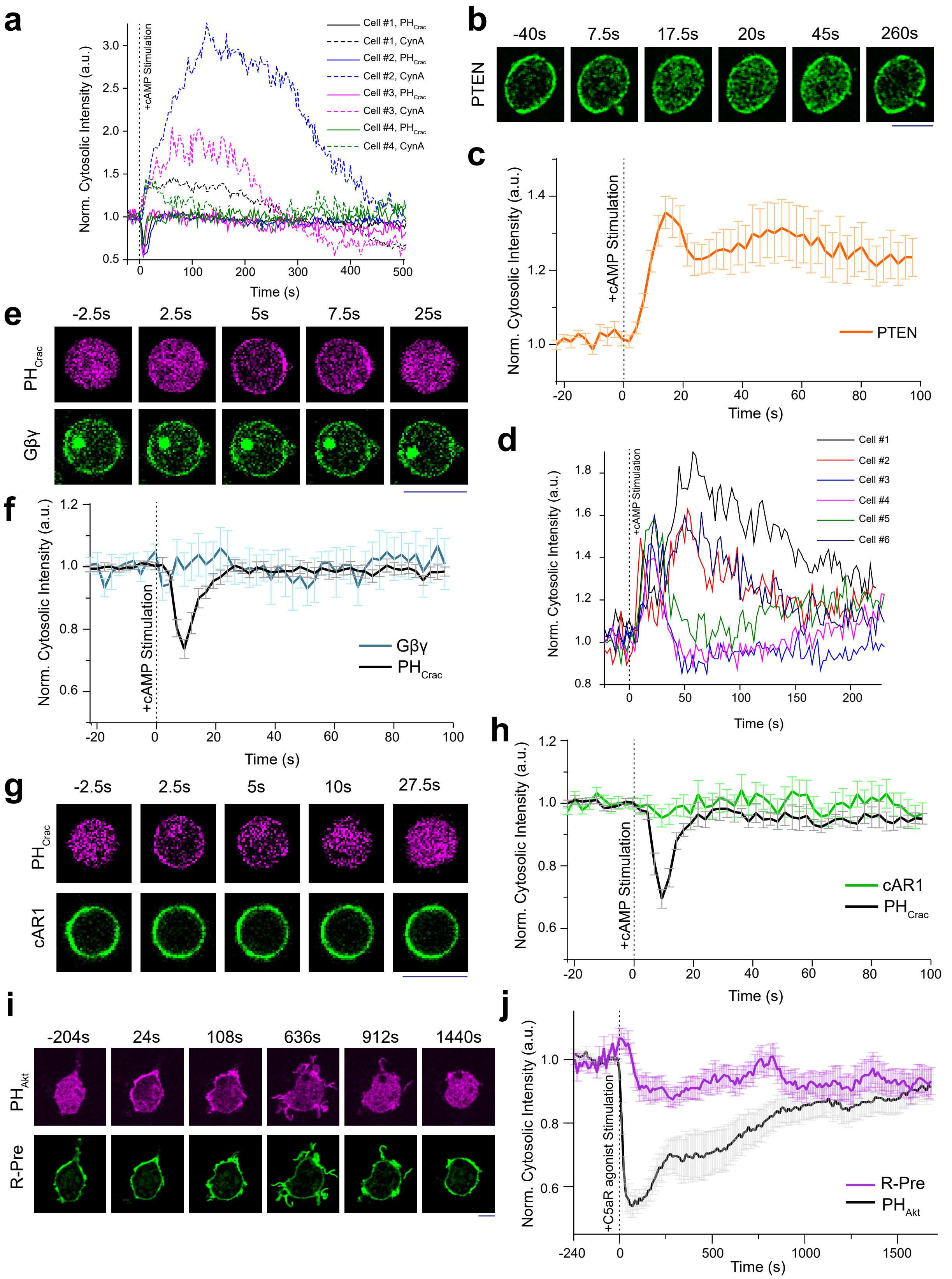

Supplementary Figure 8 | Different dissociation dynamics of different types of back-state associated membrane proteins upon global activation of receptors.

(a) Normalized cytosolic intensity profiles of 4 individual *Dictyostelium* cells co-expressing CynA-KikGR and PH_{Crac}-mCherry over 500s, upon global stimulation with cAMP. In all cases, vertical dashed lines are used to indicate the stimulation time t=0s, and at that time, 10 μM (final concentration) cAMP was added. Note that, both CynA and PH_{Crac} responses adapt over time (although CynA took longer time to adapt).

(b-d) Response of *Dictyostelium* cells expressing PTEN-GFP upon global cAMP stimulation. Live-cell time-lapse images (b), normalized cytosolic intensity profile of population (c), and normalized cytosolic intensity profile of 6 individual cells (d) are shown. Mean $\pm$ SEM are shown for n_c= 15 cells in (c).

(e) Live-cell images of *Dictyostelium* cell co-expressing PH_{Crac}-mCherry and KikGR-Gβ upon global activation of surface receptor, showing transient global recruitment of PH_{Crac} to membrane whereas G$\beta\gamma$ remained steadily membrane bound.

(f) Normalized cytosolic intensity profiles of G$\beta\gamma$ and PH_{Crac} over time, upon global stimulation with cAMP. Mean $\pm$ SEM are shown for n_c= 14 cells.

(g, h) Response of *Dictyostelium* cells co-expressing cAR1-GFP and PH_{Crac}-mCherry upon global cAMP stimulation. Live-cell images (g) and temporal profile of normalized cytosolic intensity of cAR1 and PH_{Crac} (h) are showing transient recruitment of PH_{Crac} to membrane whereas cAR1 remained steadily membrane bound. Mean $\pm$ SEM are shown for n_c= 14 cells in (h).

(i, j) Response of RAW 264.7 macrophages co-expressing GFP-R(+8)-Pre and PH_{Akt}-mCherry upon global stimulation with C5aR agonist (to 10 μM final concentration). Live-cell images (i) and temporal profile of normalized cytosolic intensity of R(+8)-Pre and PH_{Akt} (j) are demonstrating the transient recruitment of PH_{Akt} to membrane whereas R(+8)-Pre remained consistently membrane bound. Mean $\pm$ SEM are shown for n_c= 12 cells in (j).

Source data are provided as a Source Data file.

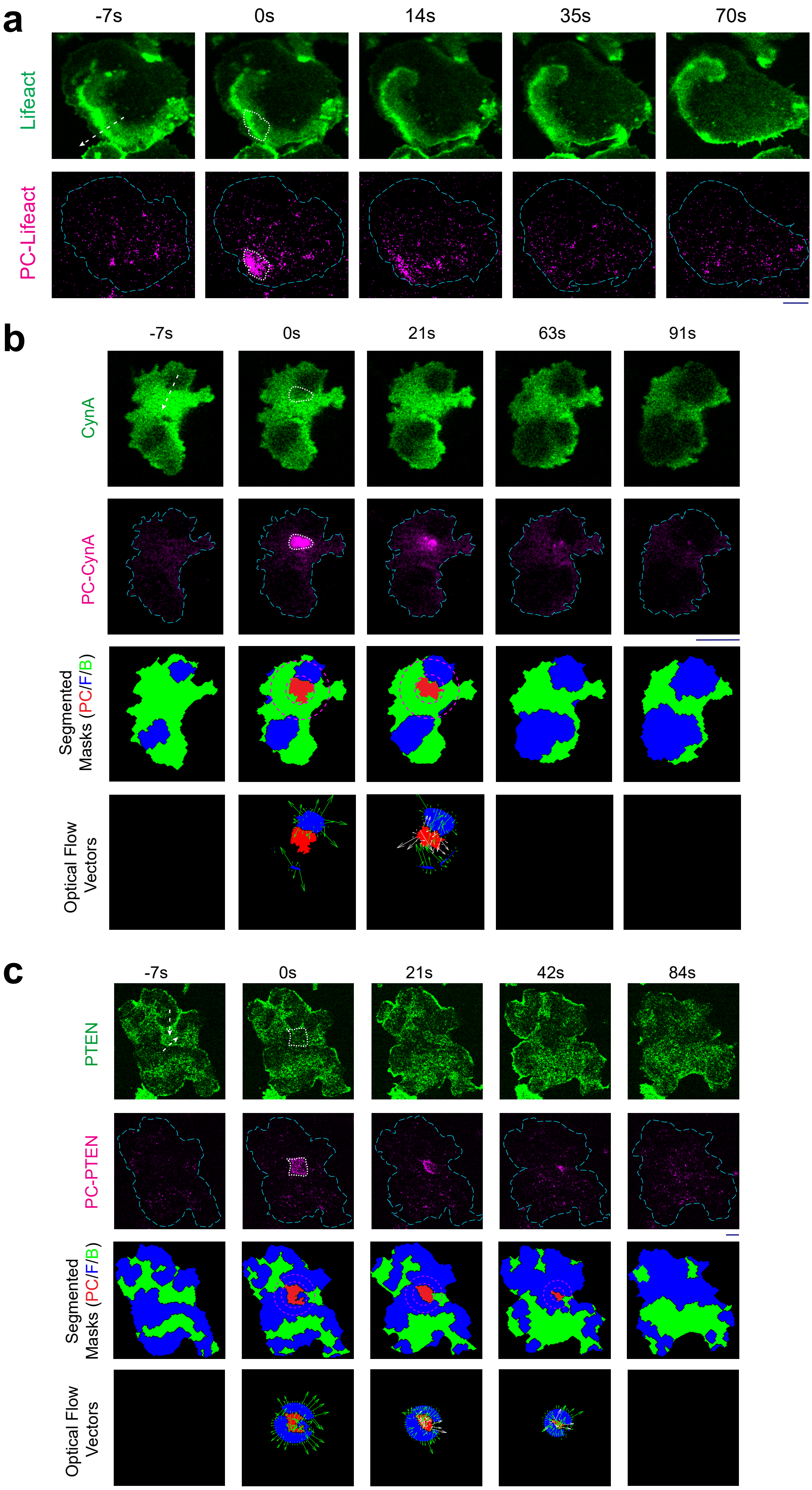
a
-7s
0s
14s
35s
70s
Lifeact
PC-Lifeact
b
-7s
0s
21s
63s
91s
CynA
PC-CynA
Segmented Masks (PC/F/B)
Optical Flow Vectors
c
-7s
0s
21s
42s
84s
PTEN
PC-PTEN
Segmented Masks (PC/F/B)
Optical Flow Vectors

Supplementary Figure 9 | Dynamics of shuttling-type peripheral membrane proteins in the photoconverted areas ahead of front/activated regions of the membrane.

(a) Live-cell time-lapse images of *Dictyostelium* cells expressing Lifeact-Dendra2 showing rapid dissociation (and resulting disappearance) of photoconverted (PC)-Lifeact molecules from the membrane as waves propagated through the initial illumination area, indicating a fast exchange of membrane-bound Lifeact with the cytosolic pool. In all cases, arrows in the first time frame in the green channel show the approximate direction of wave propagation. In all cases, enclosed white dashed areas were photoconverted with 405 nm illumination at time t=0s.
(b, c) Live-cell time-lapse images of *Dictyostelium* cells expressing CynA-KikGR (b) or PTEN-KikGR (c) showing a rapid dissociation (and resulting disappearance) of PC-CynA and PC-PTEN molecules from the membrane as front-waves propagated through the initial illumination area, indicating a fast exchange of membrane-bound CynA with the cytosolic pool. The convention used in the last two rows of horizontal panels are same as described in Figure 4c and Figure 4d.

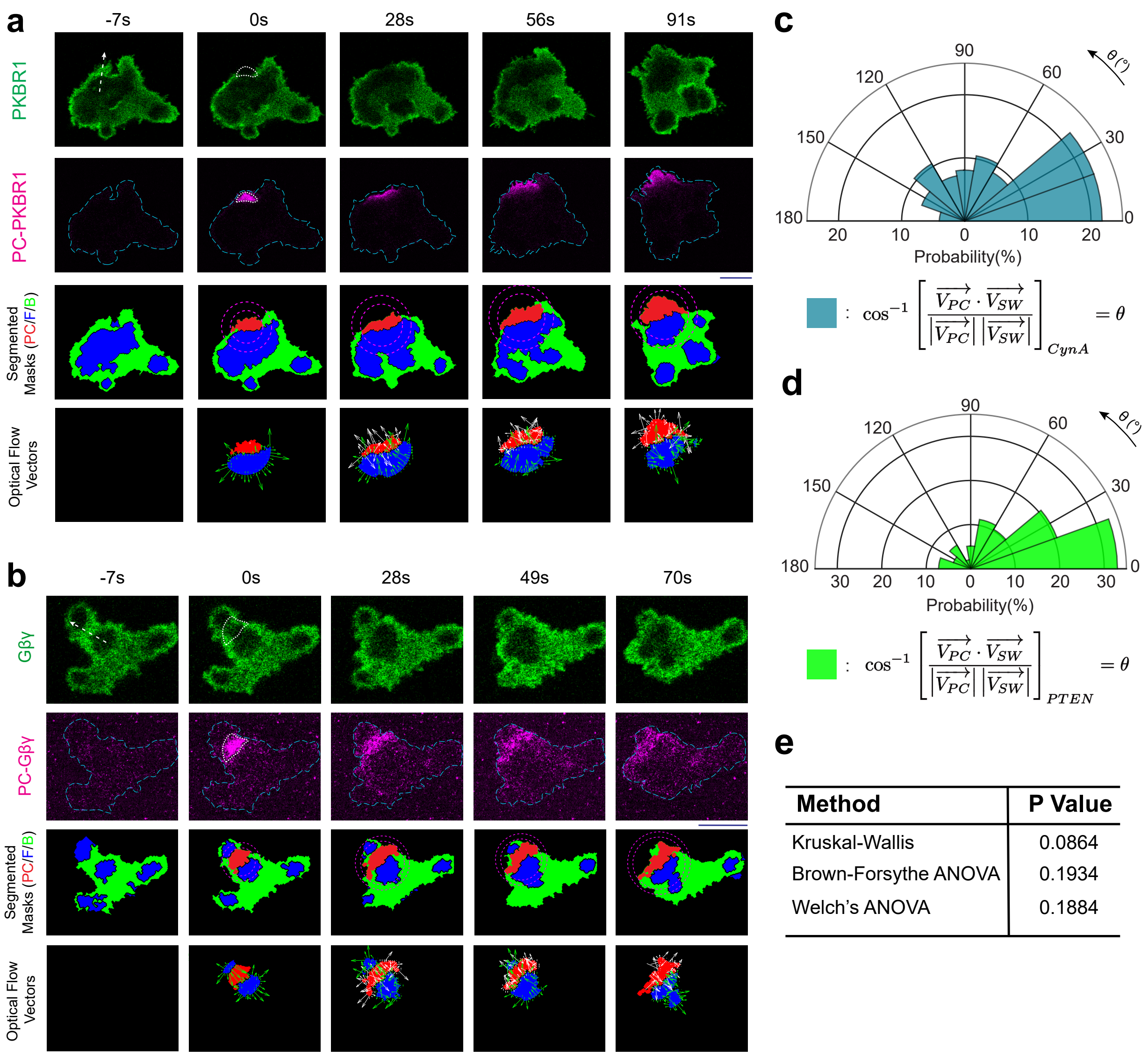

Method	P Value
Kruskal-Wallis	0.0864
Brown-Forsythe ANOVA	0.1934
Welch's ANOVA	0.1884

Supplementary Figure 10 | **Analysis of spatiotemporal dynamics of photoconverted areas right ahead of propagating front/activated-state regions of the membrane.**

(a, b) Live-cell time-lapse images of *Dictyostelium* cells expressing PKBR1-KikGR (a) or KikGR-Gβ (b) showing minimal dissociation of PC-PKBR1 and PC-G$\beta\gamma$ molecules from the membrane as waves propagated through the initial photoconversion area. The convention used in the last two horizontal panels are same as described in Figure 4c and Figure 4d.

(c, d) Polar histograms showing the probability distribution of angle between resultant optical flow vector of shadow-waves ($\overrightarrow{V_{SW}}$) and the resultant optical flow vector of photoconverted regions ($\overrightarrow{V_{PC}}$). (c) shows the distribution for CynA and (d) shows the distribution for PTEN. Both cases are indicating that the loss of intensity in the photoconverted areas of CynA and PTEN was due to the effect of the front-state waves

crossing the regions. n_f=124 frames for (c) and n_f=97 frames for (d).

(e) P-values obtained by different ANOVA statistical tests showing no difference in the distrbituion of angle between $\overrightarrow{V_{SW}}$ and $\overrightarrow{V_{PC}}$ for PKBR1, $G\beta\gamma$, PTEN, and CynA demonstrating in all cases, the intensity profiles of photoconverted molecules successfully interfered with front-wave propagation.

Source data are provided as a Source Data file.

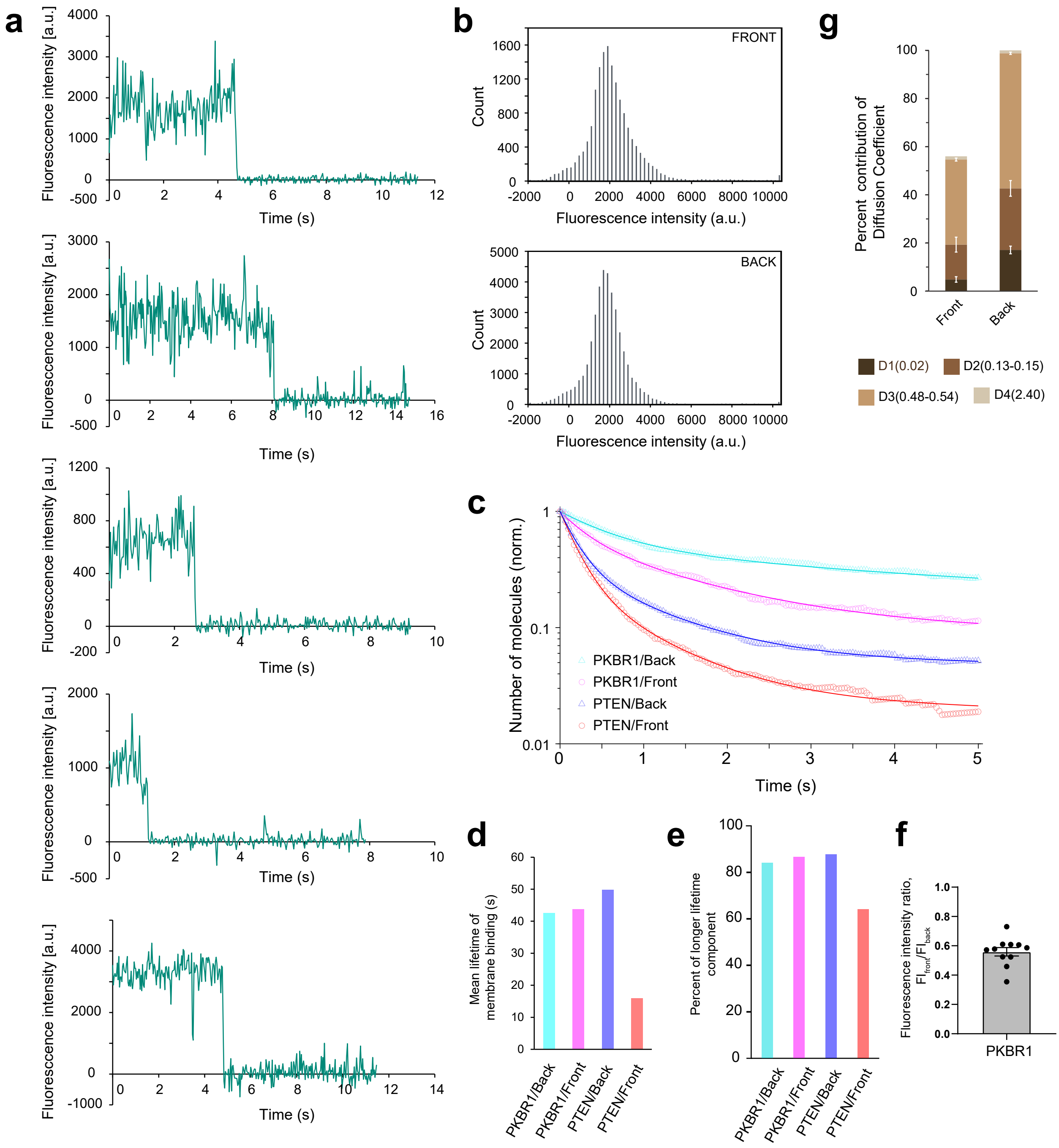

Supplementary Figure 11 | **Single molecule imaging and analysis.**

(a) Five representative time series of fluorescent intensities of TMR conjugated PKBR1-Halo demonstrating single-step photobleaching, a characteristic of single-molecule imaging.

(b) Single-peaked distribution of fluorescence intensities of PKBR1-Halo-TMR was observed in "front" as well as "back" states of the membrane during wave propagation.

(c) Temporal profile of dissociation of PKBR1 and PTEN from front (red/orange) vs back states (blue/cyan) of the membrane demonstrating that, compared to PTEN, a substantially higher fraction of PKBR1 molecules remain membrane bound during the time course of experiments. Lines represent the fitted data. Means are shown.
(d) Mean lifetime of membrane binding of PKBR1 and PTEN molecules in front and back states of the membrane. Note that the mean lifetimes of PKBR1 molecules at the front- and back-state membranes were 43.7 s and 42.6 s, while those of PTEN molecules were 16.0 s and 49.8 s at the front- and back-state membranes, respectively, demonstrating that the mean lifetime of PKBR1 was not affected by the membrane state, while that of PTEN significantly shortened in the front membranes.
(e) Fraction of the longer lifetime molecules of PKBR1 and PTEN in front and back states (as obtained by the single-molecule imaging) of the membrane demonstrating that the mean lifetime is roughly dependent on the fraction of the longest component except for PTEN at the front-state membrane, where the lifetime as well as the fraction of the longest component were reduced, leading to the reduction of the mean lifetime to about 0.3-fold of that of the back-state (also see Supplementary Figure 11d and Supplementary Table 1). Note that, the shorter two components were detected more frequently than this longer component due to their quick turn-over behaviors and affected the initial decay of the curves, but they had little influence on the mean lifetime since these fractions in the total membrane-bound molecules were small.
(f) Ratio of fluorescence intensity of TMR conjugated PKBR1-Halo at the front-state membrane compared to the back-state membrane, as measured in the TIRFM images (n=11 cells, $mean \pm SEM$ and data points are shown).
(g) Different diffusion coefficients and their fraction, corresponding to different mobility states of PKBR1 molecules, associated with either front or back-state regions of the membrane. The distribution of data (as shown in Figure 5g) was fitted to compute the diffusion coefficients. Error bars showing 95% confidence interval.
Source data are provided as a Source Data file.

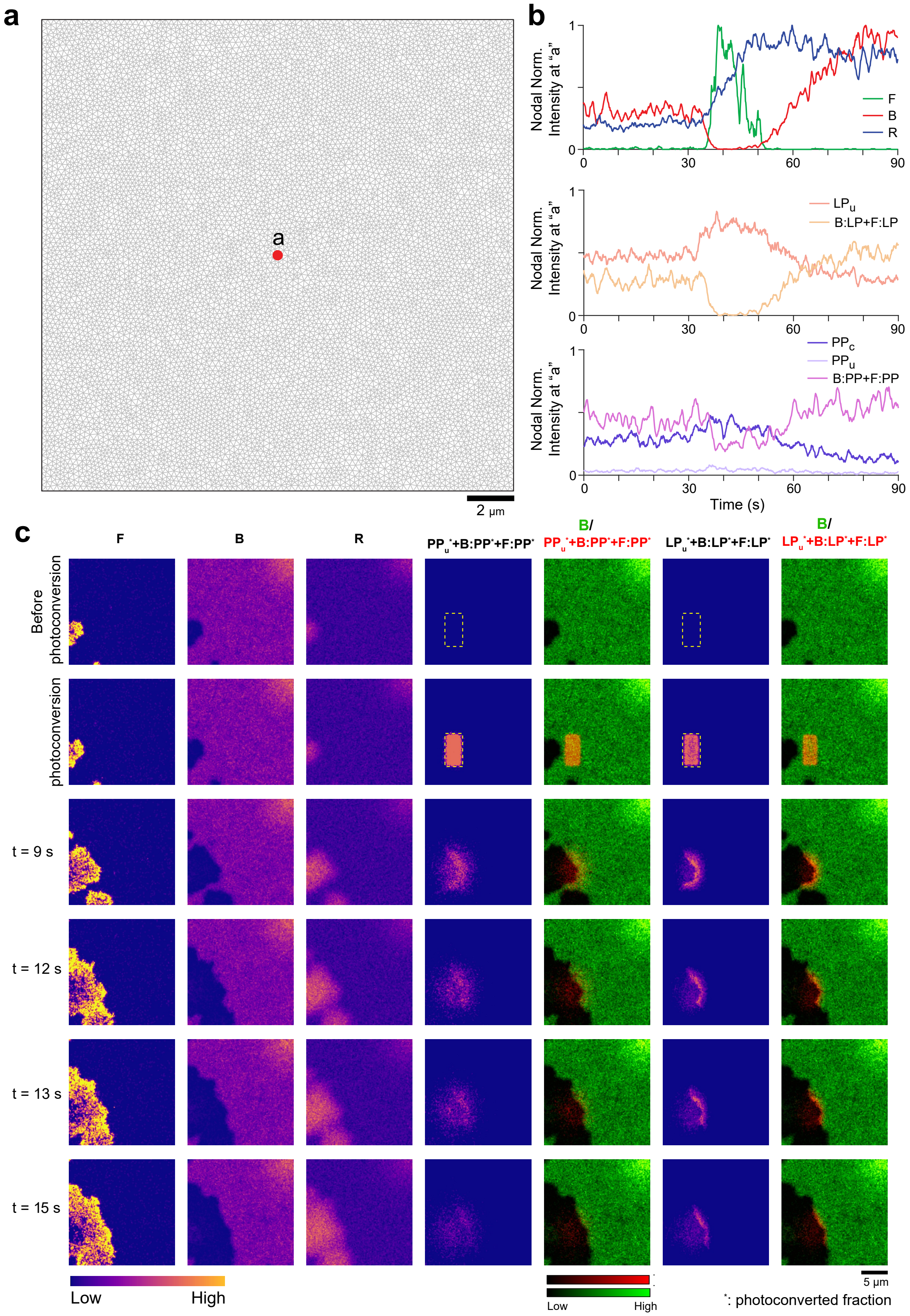
a
b
c
2 µm
Nodal Norm. Intensity at "a"
F
B
R
LP$_u$
B:LP+F:LP
PP$_c$
PP$_u$
B:PP+F:PP
Time (s)
Before photoconversion
photoconversion
t = 9 s
t = 12 s
t = 13 s
t = 15 s
PP$_u^*$+B:PP*+F:PP*
B/ PP$_u^*$+B:PP*+F:PP*
LP$_u^*$+B:LP*+F:LP*
B/ LP$_u^*$+B:LP*+F:LP*
Low
High
5 µm
*: photoconverted fraction

Supplementary Figure 12|**Simulated local temporal profiles of different species and spatiotemporal dynamics of different classes of membrane proteins during *in silico* photoconversion.**

(a) Mesh of the square-shaped simulation domain ($20\mu m \times 20\mu m$) used in the present study. The maximum nodal distance was chosen to be $0.25\mu m$ resulting 11146 nodes in total.

(b) Temporal profiles of normalized nodal intensity at node "a" (as shown in (a)) for different species with their respective cytosolic fractions (denoted with "c" subscript); membrane-associated, freely moving unbound fractions (denoted with "u" subscript), and slowly moving membrane-bound fractions (denoted with B: and F: notations, for back-region bound and front-region bound sub-fractions, respectively).

(c) Simulated spatiotemporal profiles of F, B, and photoconverted total membrane fractions of PP and LP. A rectangular section (yellow dashed in first and second row) in front of the propagating wave was selected for photoconversion where all the molecules of LP and PP on the membrane was converted to respective photoconverted forms (denoted with "*" superscript). As the wave hits the photoconverted region (i.e. B-state started switching to F-state in that spatial domain), photoconverted membrane-associated PP molecules ($PP_u^* + B : PP^* + F : PP^*$) disappeared fast from that region (primarily due to shuttling to cytosol), whereas the photoconverted membrane-associated LP molecules ($LP_u^* + B : LP^* + F : LP^*$) stayed on the membrane for longer time.

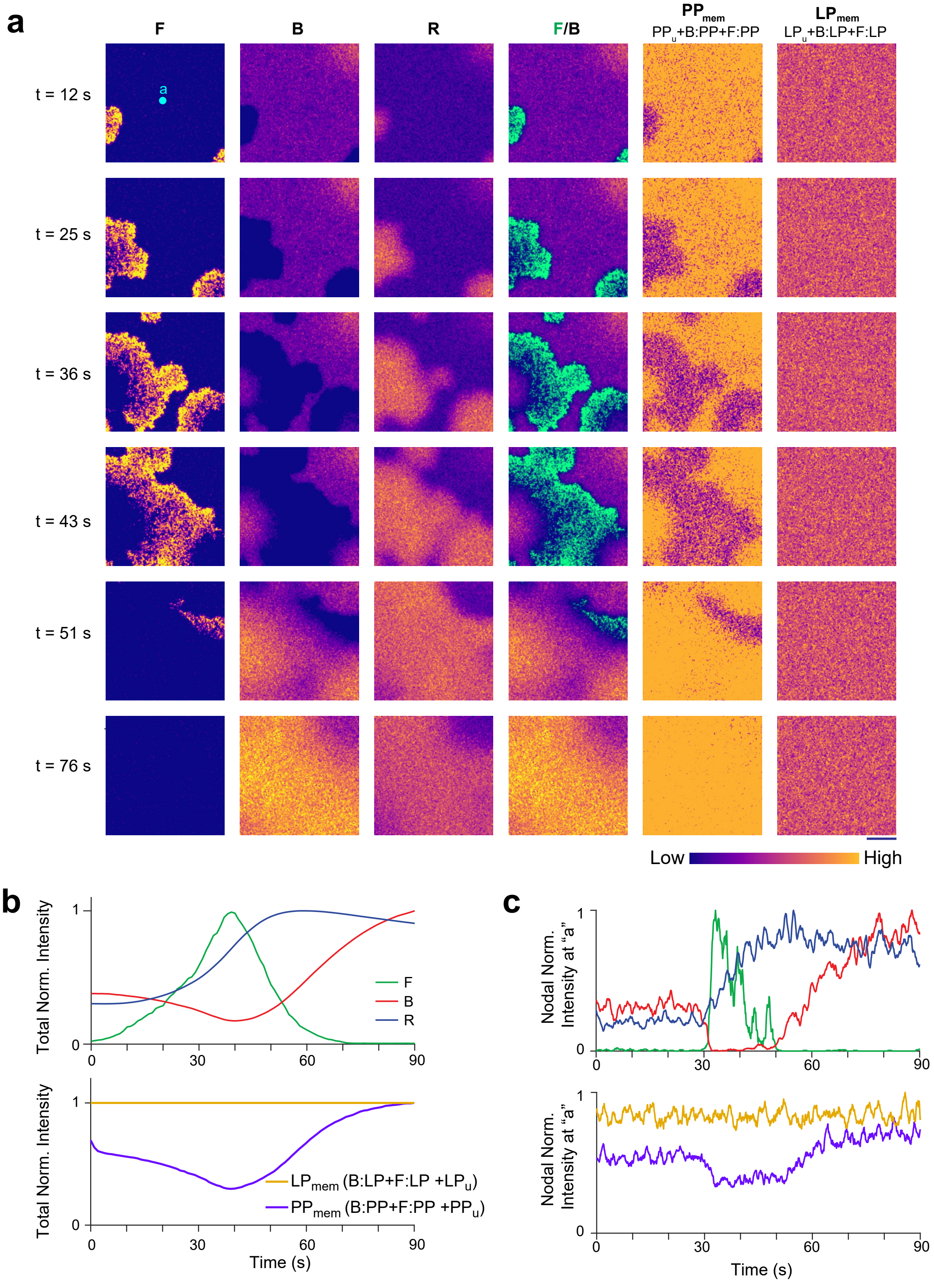

Supplementary Figure 13 | Stochastic spatiotemporal simulation to assess the effects of differences in the diffusion coefficients of the different membrane associated states of the lipid-anchored proteins.

(a) Simulated spatiotemporal profiles of different species (F, R, B, total PP on membrane, and total LP). Here, instead of using experimentally found values, the diffusion coefficients of bound and unbound states of LP are assumed to be equal (see Supplementary Table 4). Note that, due to lack of difference in diffusion coefficient, during wave propagation of the excitable network, LP could not exhibit any compartmentalization whereas the compartmentalization behavior of PP remained unchanged. Subscripts follow the same notation as Figure 6b and 6c.
(b, c) Temporal profiles of normalized total intensity (b) and normalized nodal intensity at node “a” (which is shown as a cyan dot in (a)) (c), for different species. Same line colors for each species were used in (b) and (c) plots.

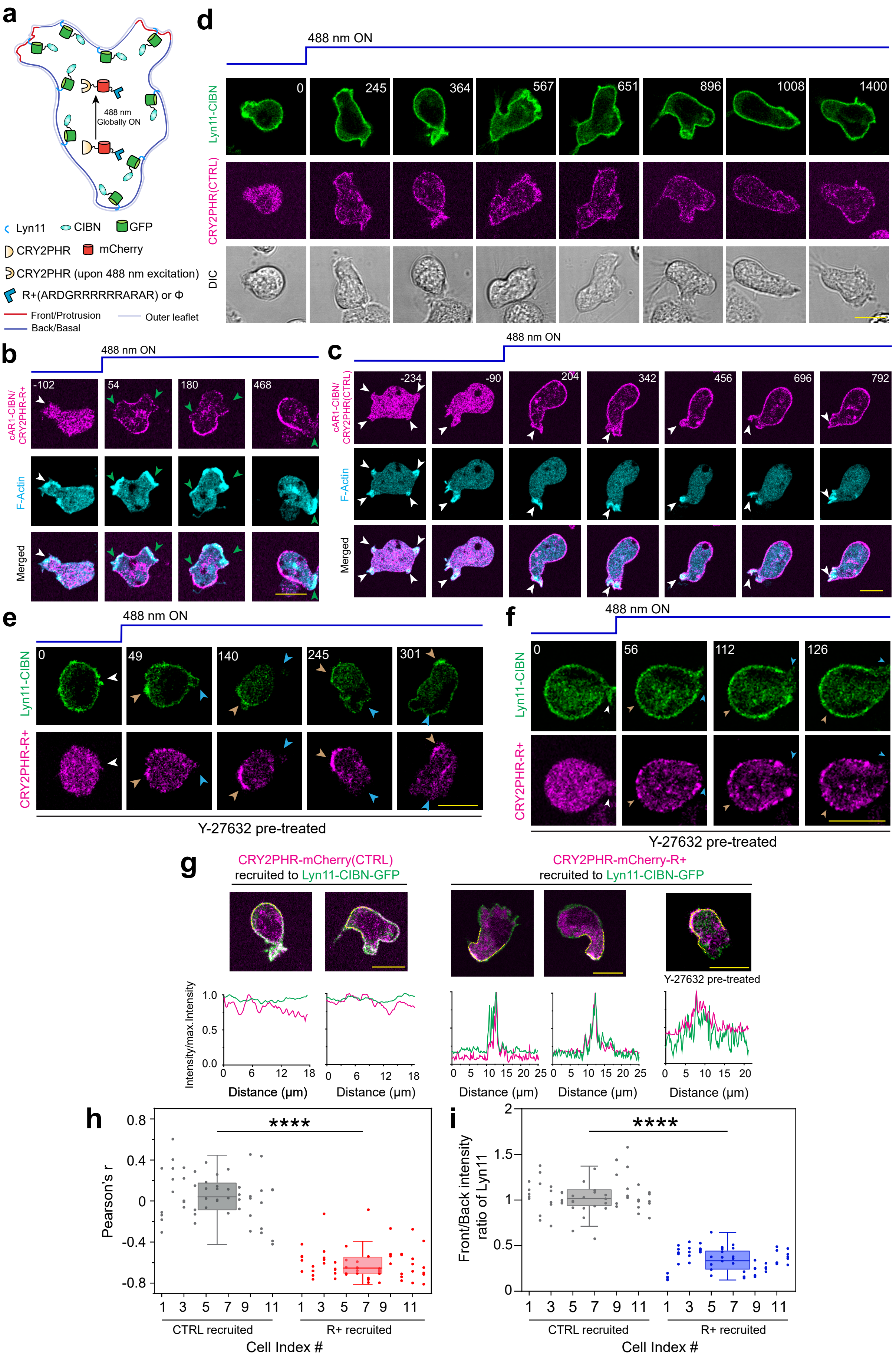
a
488 nm Globally ON
Lyn11
CIBN
GFP
CRY2PHR
mCherry
CRY2PHR (upon 488 nm excitation)
R+(ARDGRRRRRRARAR) or Φ
Front/Protrusion
Back/Basal
Outer leaflet
d
488 nm ON
Lyn11-CIBN
CRY2PHR(CTRL)
DIC
0
245
364
567
651
896
1008
1400
b
488 nm ON
cAR1-CIBN/ CRY2PHR-R+
F-Actin
Merged
-102
54
180
468
c
488 nm ON
cAR1-CIBN/ CRY2PHR(CTRL)
F-Actin
Merged
-234
-90
204
342
456
696
792
e
488 nm ON
Lyn11-CIBN
CRY2PHR-R+
0
49
140
245
301
Y-27632 pre-treated
f
488 nm ON
Lyn11-CIBN
CRY2PHR-R+
0
56
112
126
Y-27632 pre-treated
g
CRY2PHR-mCherry(CTRL) recruited to Lyn11-CIBN-GFP
CRY2PHR-mCherry-R+ recruited to Lyn11-CIBN-GFP
Y-27632 pre-treated
Intensity/max.Intensity
Distance (μm)
h
Pearson's r
****
CTRL recruited
R+ recruited
Cell Index #
i
Front/Back intensity ratio of Lyn11
****
CTRL recruited
R+ recruited
Cell Index #

Supplementary Figure 14 | Optogenetic recruitment of positively charged and uncharged peptide to uniformly distributed lipidated and integral membrane proteins.

(a) Schematic for selectively increasing the membrane back-state region or uropod specific affinity of uniformly distributed lipid-anchored (myristoylated and palmitoylated) protein Lyn11 in neutrophils. Upon 488 nm laser irradiation, the cytosolic CRY2PHR that is fused with positively charged peptide(R+) or blank (ϕ) (shown as a blue hexagon), gets globally recruited to CIBN fused Lyn11 on membrane.

(b) Live-cell images of *Dictyostelium* cell co-expressing cAR1-CIBN, CRY2PHR-mCherry-R+, and Lifeact-HaloTag(Janelia Fluor 646), before and after recruitment of CRY2PHR-mCherry-R+ with 488 nm laser excitation, demonstrating that, upon recruitment, CRY2PHR-mCherry-R+/cAR1-CIBN was dynamically excluded from the protrusions. White arrowheads: F-actin rich protrusions before recruitment or cAR1 polarization; Green arrowheads: F-actin rich protrusions from where cAR1-CIBN/CRY2PHR-mcherry-R+ was absent.

(c) Representative live-cell images of *Dictyostelium* cell co-expressiing cAR1-CIBN, CRY2PHR-mCherry(CTRL), and Lifeact-HaloTag(Janelia Fluor 646), showing global optogenetic recruitment of CRY2PHR-mCherry(CTRL) from cytosol to membrane did not induce asymmetric distribution of cAR1. White arrowheads: F-actin rich protrusions, where cAR1 was also present.

(d) Representative live-cell images of differentiated HL-60 neutrophil cells, co-expressing cytosolic CRY2PHR-mCherry(CTRL) and membrane bound Lyn11-CIBN-GFP. Note that optogenetic global recruitment of CRY2PHR-mCherry(CTRL) from cytosol to membrane did not result in any symmetry breaking of Lyn11 over membrane.

(e, f) Two representative examples of live-cell time-lapse image series of differentiated HL-60 neutrophil cells, co-expressing cytosolic CRY2PHR-mCherry-R+ and membrane bound Lyn11-CIBN-GFP, before and after light-induced recruitment. Cells were pre-treated with ROCK inhibitor Y-27632 before starting the imaging experiment. In (e) and (f), white arrowheads: Protrusions before starting the recruitment; Blue arrowheads: Protrusions after recruitment (which is depleted of both Lyn11-CIBN-GFP and CRY2PHR-mCherry-R+); Teal arrowheads: Back-state regions of the cell membrane after recruitment (where both Lyn11-CIBN-GFP and CRY2PHR-mCherry-R+ are selectively enriched).

(g) Line-scan analysis of intensity profiles of Lyn11-CIBN-GFP and recruited CRY2PHR-mCherry(CTRL) or CRY2PHR-mCherry-R+ (untreated and Y-27632 pre-treated), along the dotted yellow lines on the membrane, demonstrating that in contrast to CRY2PHR-mCherry(CTRL), the recruitment of CRY2PHR-mCherry-R+ to the membrane, presumably due to its positive charge (which targets highly negatively charged back-regions of membrane), induce partitioning to selectively align Lyn11 to the uropods or back-regions of the membrane in differentiated HL-60 cells.

(h) Box and whisker plots and aligned dot plots of Pearson's correlation coefficients between front-state marker LimE and CRY2PHR-mCherry-R+/cAR1-CIBN or CRY2PHR-mCherry(CTRL)/cAR1-CIBN. The x-axis is showing index number for each cell in aligned dot plot. Pearson's r values were calculated along the membrane after the recruitment. For each of the $n_c = 11$ (for CTRL) or $n_c = 12$ (for R+) cells, Pearson's r values for $n_f = 5$ frames were plotted. The p-values by Mann-Whitney-Wilcoxon test.
(i) Box and whisker plots and aligned dot plots of front to back intensity ratio of Lyn11, after the recruitment of CRY2PHR-mCherry-R+ or CRY2PHR-mCherry(CTRL), in differentiated HL-60 neutrophil cells. For each of the $n_c = 12$ (for CTRL) and $n_c = 12$ (for R+) cells, intensity ratio values for $n_f = 5$ frames were plotted. The p-values by Mann-Whitney-Wilcoxon test.
Source data are provided as a Source Data file.

Supplementary Table 1 | Lifetimes of PKBR1 and PTEN on the front- and back-state regions of the membranes.

		PKBR1		PTEN	
		Back	Front	Back	Front
**Faster exponential component**					
a_1		0.51	0.43	0.66	0.76
		(0.48~0.55)	(0.27~0.58)	(0.65~0.68)	(0.71~0.80)
k_1	[/sec]	1.75	3.33	4.90	4.96
		(1.68~1.83)	(2.62~4.04)	(4.77~5.02)	(4.73~5.20)
τ_1*	[sec]	0.64	0.32	0.21	0.21
$a_1 \tau_1$*		0.33	0.14	0.14	0.16
fraction		0.03	0.02	0.04	0.16
**Intermediate exponential component**					
a_2		0.30	0.47	0.28	0.22
		(0.16~0.45)	(0.36~0.58)	(0.27~0.30)	(0.18~0.26)
k_2	[/sec]	0.42	0.90	1.14	1.29
		(0.29~0.55)	(0.35~1.46)	(1.08~1.21)	(1.06~1.53)
τ_2*	[sec]	4.51	1.42	1.00	0.87
$a_2 \tau_2$*		1.37	0.67	0.28	0.19
fraction		0.13	0.11	0.08	0.20
**Slower exponential component**					
a_3		0.18	0.10	0.05	0.03
k_3	[/sec]	0.22	0.22	0.16	0.18
		(0.17~0.27)	(-0.16~0.60)	(0.15~0.17)	(0.11~0.25)
τ_3*	[sec]	50.0	50.0	56.5	24.7
$a_3 \tau_3$*		9.13	5.22	3.05	0.62
fraction		0.84	0.87	0.88	0.64
**Mean lifetime**					
	[sec]	42.6	43.7	49.8	16.0

Values written in the brackets denote the 95% confidence intervals.
*Values are corrected for the fluorophore photobleaching.

Supplementary Table 2 | Diffusion coefficients of PKBR1 on the front- and back-state regions of the membranes.

		PKBR1	
		Back	Front
**Diffusion coefficients (4-state model)**			
D_1	[μm^2/sec]	0.017	0.019
	95% CI	0.016~0.019	0.015~0.023
	fraction	0.17	0.09 (0.05)
	95% CI	0.16~0.19	0.07~0.11
D_2	[μm^2/sec]	0.13	0.15
	95% CI	0.10~0.15	0.11~0.19
	fraction	0.26	0.26 (0.15)
	95% CI	0.22~0.29	0.20~0.31
D_3	[μm^2/sec]	0.48	0.54
	95% CI	0.45~0.50	0.50~0.58
	fraction	0.56	0.63 (0.35)
D_4	[μm^2/sec]	2.45	2.39
	95% CI	1.95~2.94	1.82~2.97
	fraction	0.01	0.02 (0.01)
	95% CI	0.01~0.02	0.01~0.04
**Akaike information criterion [maximum log-likelihood]**			
1-state model [k=1]		-53880 [26941.1]	-19590 [9795.9]
2-state model [k=3]		-64753 [32379.6]	-21944 [10975.0]
3-state model [k=5]		-65990 [33000.0]	-22470 [11239.8]
4-state model [k=7]		-66263 [33138.5]	-22562 [11288.1]
5-state model [k=9]		-66258 [33138.1]	-22557 [11287.7]
6-state model [k=11]		-66249 [33135.6]	-22553 [11287.7]

Fractions written in the parentheses denote the values after corrected for the total amount of molecules which was smaller at the front-state membrane than back. For front state, 15386 data points and for back-state 38641 data points were analyzed. The diffusion coefficients and their fractions were estimated by maximum likelihood estimation on MATLAB using `mle` or `fmincon`, which provided essentially the same estimates. The 95% CI was calculated with `mle`. AIC was calculated using $AIC = -2ln(L) + 2k$, where $ln(L)$ and k denote the maximum log-likelihood and the number of parameters, respectively. The ln(L) was obtained by finding the minimum of -ln(L) with `fmincon`.

Supplementary Table 3 | Determining diffusion coefficients for computational simulations.

	**1 state**	**2 states**	**3 states**	**4 states**	**5 states**
D_1	0.42	0.05	0.01	0.01	0.01
D_2		0.45	0.16	0.16	0.16
D_3			0.48	0.27	0.48
D_4				0.48	11.64
D_5					46.67
k	3	6	9	12	15
AIC	-701.04	-784.99	-793.20	-787.21	-781.20
AICc	-700.72	-783.83	-790.63	-782.55	-773.70
p_1 (back)	1	0.40	0.23	0.25	0.23
p_2 (back)		0.60	0.21	0.13	0.21
p_3 (back)			0.55	0.15	0.55
p_4 (back)				0.47	9.56×10^{-13}
p_5 (back)					4.80×10^{-16}
p_1 (front)	1	0.18	0.06	0.06	0.06
p_2 (front)		0.82	0.29	0.29	0.29
p_3 (front)			0.64	0	0.64
p_4 (front)				0.64	0.00
p_5 (front)					0.00

The AIC values were computed using the formula: $AIC = nlog\left(\frac{SSE}{n}\right) + 2k$. The AIC corrected (AICc), which was developed to avoid potential overfitting for small sample size (especially in cases where $\frac{n}{k} < 40$) values, were computed using the formula: $AICc = AIC + \frac{2k(k+1)}{n-k-1}$. Here, n is sample size (n=80 for our case), k is number of parameters, and SSE is sum of squared estimate of errors. Please see the *Computational modelling* subsection B (in Methods section) for further details.

Supplementary Table 4 | Parameters used in the stochastic simulations.

No.	Reaction	Propensity	Stoichiometry Vector: F	B	R	LP_u	B:LP	F:LP	PP_c	PP_u	B:PP	F:PP	Parameter	Nom. Val.	Units
1.	$F \rightarrow \varnothing$	$a_1[F]$	−1	0	0	0	0	0	0	0	0	0	a_1	1.66	$\times 10^{-2} s^{-1}$
2.	$F \xrightarrow{R} \varnothing$	$a_2[F][R]$	−1	0	0	0	0	0	0	0	0	0	a_2	33.32	$[\mu M\, s]^{-1}$
3.	$\varnothing \xrightarrow{B\perp} F$	$\frac{a_3(a_5 - F)}{a_4^2[B][B-1]+1}$	+1	0	0	0	0	0	0	0	0	0	a_3	18.74	s^{-1}
													a_4	2880	μM^{-1}
													a_5	2	μM
4.	$\varnothing \rightarrow F$	$a_6(a_5 - [F])$	+1	0	0	0	0	0	0	0	0	0	a_6	2.94	$\times 10^{-2} s^{-1}$
5.	$\varnothing \rightarrow B$	b_1	0	+1	0	0	0	0	0	0	0	0	b_1	0.1	$\mu M s^{-1}$
6.	$B \rightarrow \varnothing$	$b_2[B]$	0	−1	0	0	0	0	0	0	0	0	b_2	2	$\times 10^{-3} s^{-1}$
7.	$B \xrightarrow{F} \varnothing$	$b_3[B][F]$	0	−1	0	0	0	0	0	0	0	0	b_3	40	$[\mu M\, s]^{-1}$
8.	$R \rightarrow \varnothing$	$c_1[R]$	0	0	−1	0	0	0	0	0	0	0	c_1	4	$\times 10^{-3} s^{-1}$
9.	$\varnothing \xrightarrow{F} R$	$c_2[F]$	0	0	+1	0	0	0	0	0	0	0	c_2	25.6	$\times 10^{-2} s^{-1}$
10.	$LP_u \xrightarrow{B} B{:}LP$	$d_1[B][LP_u]$	0	0	0	−1	+1	0	0	0	0	0	d_1	124.4	$[\mu M\, s]^{-1}$
11.	$B{:}LP \rightarrow LP_u$	$d_2[B{:}LP]$	0	0	0	+1	−1	0	0	0	0	0	d_2	108	s^{-1}
12.	$LP_u \xrightarrow{F} F{:}LP$	$d_3[F][LP_u]$	0	0	0	−1	0	+1	0	0	0	0	d_3	0.05	$[\mu M\, s]^{-1}$
13.	$F{:}LP \rightarrow LP_u$	$d_4[F{:}LP]$	0	0	0	+1	0	−1	0	0	0	0	d_4	24	s^{-1}
14.	$PP_c \rightarrow PP_u$	$e_1[PP_c]$	0	0	0	0	0	0	−1	+1	0	0	e_1	1	s^{-1}
15.	$PP_u \rightarrow PP_c$	$e_2[PP_u]$	0	0	0	0	0	0	+1	−1	0	0	e_2	8	s^{-1}
16.	$PP_u \xrightarrow{B} B{:}PP$	$e_3[B][PP]$	0	0	0	0	0	0	0	−1	+1	0	e_3	17	$[\mu M\, s]^{-1}$
17.	$B{:}PP \rightarrow PP_u$	$e_4[B{:}PP]$	0	0	0	0	0	0	0	+1	−1	0	e_4	0.8	s^{-1}
18.	$PP_u \xrightarrow{F} F{:}PP$	$e_3[F][PP]$	0	0	0	0	0	0	0	−1	0	+1	e_3	1.7	$[\mu M\, s]^{-1}$
19.	$B{:}PP \rightarrow PP_u$	$e_5[B{:}PP]$	0	0	0	0	0	0	0	+1	0	−1	e_4	0.08	s^{-1}

	Unit	F	B	R	LP_u	B:LP	F:LP	PP_c	B:PP	F:PP
**Diffusion constant**	$\mu m^2 s^{-1} \times 10^{-2}$	15	7.5	9	45	5	5	75	45	5
