# Supplementary material for: A dynamic partitioning mechanism polarizes membrane protein distribution
Source: Nat Commun. 2023 Nov 30;14:7909. doi: 10.1038/s41467-023-43615-2 (PMC10689845; doi:10.1038/s41467-023-43615-2)
Supplement: Supplementary file 3 — Description of Additional Supplementary Files [file 41467_2023_43615_MOESM3_ESM.pdf]

Description of Additional Supplementary Files

File Name: Supplementary Movie 1
Description: Consistent dynamic localization of PKBR1 into the back-state regions of the membrane. Ventral wave propagation in the substrate-attached surface of *Dictyostelium* cells co-expressing PKBR1-KikGR and PH_{Crac}-mCherry, demonstrating that complementary distribution between front-state marker PH_{Crac} and PKBR1 is highly consistent during dynamic pattern formations. Left Panels: PKBR1-KikGR, middle panels: PH_{Crac}-mCherry, right panels: merged view. Top right-corner: time in mm:ss format.

File Name: Supplementary Movie 2
Description: Consistent dynamic localization of Gβγ into the back-state regions of the membrane. Ventral wave propagation in the substrate-attached surface of *Dictyostelium* cells co-expressing KikGR-Gβγ and PH_{Crac}-mCherry, demonstrating that complementary distribution between front-state marker PH_{Crac} and Gβγ is highly consistent during dynamic pattern formations. Left Panels: KikGR-Gβγ, middle panels: PH_{Crac}-mCherry, right panels: merged view. Top right-corner: time in mm:ss format.

File Name: Supplementary Movie 3
Description: Consistent dynamic localization of RasG into the back-state regions of the membrane. Ventral wave propagation in the substrate-attached surface of *Dictyostelium* cells co-expressing GFP-RasG and PH_{Crac}-mCherry, demonstrating that complementary distribution between front-state marker PH_{Crac} and RasG is consistent during dynamic pattern formations. Left Panels: GFP-RasG, middle panels: PH_{Crac}-mCherry, right panels: merged view. Top right-corner: time in mm:ss format.

File Name: Supplementary Movie 4
Description: Consistent dynamic localization of synthetic protein $PKBR1_{N150}$ into the back-state regions of the membrane. Ventral wave propagation in the substrate-attached surface of *Dictyostelium* cells co-expressing $PKBR1_{N150}$-KikGR and PH_{Crac}-mCherry, demonstrating that complementary distribution between front-state marker PH_{Crac} and $PKBR1_{N150}$ is highly consistent during dynamic pattern formations. Left Panels: PKBR1N150-KikGR, middle panels: PH_{Crac}-mCherry, right panels: merged view. Top right-corner: time in mm:ss format.

File Name: Supplementary Movie 5

Description: Consistent localization of PKBR1 into the back of the membrane in chemotaxing *Dictyostelium* cells. Developed *Dictyostelium* cells, co-expressing PKBR1-KikGR and LimE-mCherry, chemotaxing towards a micropipette filled with 10 µM of cAMP. Left Panels: PKBR1-KikGR, middle panels: LimE-mCherry, right panels: DIC images (which also shows the position of micropipette). Fluorescent channels are shown in matplotlib "Plasma" colormap. Top right-corner: time in mm:ss format. Scale bar: 10 µm.

File Name: Supplementary Movie 6
Description: Uniform distribution of cAR1 on the membrane during ventral wave propagation. Ventral wave propagation in the substrate-attached surface of *Dictyostelium* cells co-expressing cAR1-GFP and PH_{Crac}-mCherry, demonstrating that cAR1 does not exhibit symmetry breaking and is uniformly distributed on the membrane. Left Panels: cAR1-GFP, middle panels: PH_{Crac}-mCherry, right panels: merged view. Top right-corner: time in mm:ss format.

File Name: Supplementary Movie 7
Description: Dynamic localization of R(+8)-Pre into the back-state regions of the membrane in RAW 264.7 macrophages. Ventral wave propagation in the substrate-attached surface of RAW 264.7 macrophages co-expressing GFP-R(+8)-Pre and PH_{Akt}-mCherry, demonstrating that complementary distribution between front-state marker PH_{Akt} and R(+8)-Pre is highly consistent during dynamic pattern formations which were induced by frustrated phagocytosis and osmotic shock. Left panels: GFP-R(+8)-Pre, middle panels: PH_{Akt}-mCherry, right panels: merged view. Top right-corner: time in mm:ss format.

File Name: Supplementary Movie 8
Description: Dynamic localization of R(+8)-Pre into the back-state regions of the membrane in cytoskeletal dynamics impaired RAW 264.7 macrophages. Ventral wave propagation in the substrate-attached surface of RAW 264.7 macrophages co-expressing GFP-R(+8)-Pre and PH_{Akt}-mCherry, demonstrating that complementary distribution between front-state marker PH_{Akt} and R(+8)-Pre is highly consistent during dynamic pattern formations (i.e., in both larger-scale waves and smaller-scale wavelets), even when actin polymerization was inhibited with 5µM Latrunculin A and ROCK activity was inhibited with 50µM of Y-27632 (se Methods for details). Left panels: GFP-R(+8)-Pre, middle panels: PH_{Akt}-mCherry, right panels: merged view. Top right-corner: time in mm:ss format.

File Name: Supplementary Movie 9
Description: Kinetics of CynA and PH_{Crac} during global receptor activation. Global cAMP stimulation driven receptor activation in *Dictyostelium* cells co-expressing CynA-KikGR and

PH_{Crac}-mCherry, demonstrating that upon receptor activation front protein PH_{Crac} gets recruited to membrane from cytosol whereas back-associated peripheral membrane protein CynA gets dissociated from membrane and moves to cytosol. Left Panels: CynA-KikGR, right panels: PH_{Crac}-mCherry. Top right-corner: time in seconds. cAMP was added at time t=0s (also indicated by the appearance of white text “+cAMP stimulation” in the video).

File Name: Supplementary Movie 10
Description: Kinetics of PKBR1 and PH_{Crac} during global receptor activation. Global cAMP stimulation driven receptor activation in *Dictyostelium* cells co-expressing PKBR1-KikGR and PH_{Crac}-mCherry, demonstrating that upon receptor activation front protein PH_{Crac} gets recruited to membrane from cytosol whereas back-associated lipid-anchored protein PKBR1 maintained membrane association. Left Panels: PKBR1-KikGR, right panels: PH_{Crac}-mCherry. Top left-corner: time in seconds. cAMP was added at time t=0s (also indicated by the appearance of white text “+cAMP stimulation” in the video).

File Name: Supplementary Movie 11
Description: Kinetics of Gβγ and PH_{Crac} during global receptor activation. Global cAMP stimulation driven receptor activation in *Dictyostelium* cells co-expressing KikGR-Gβγ and PH_{Crac}-mCherry, demonstrating that upon receptor activation front protein PHCrac gets recruited to membrane from cytosol whereas back-associated lipid-anchored protein Gβγ maintained membrane association. Left Panels: KikGR-Gβγ, right panels: PH_{Crac}-mCherry. Top left-corner: time in seconds. cAMP was added at time t=0s (also indicated by the appearance of white text “+cAMP stimulation” in the video).

File Name: Supplementary Movie 12
Description: Kinetics of R(+8)-Pre and PH_{Crac} during global receptor activation. Global cAMP stimulation driven receptor activation in *Dictyostelium* cells co-expressing GFP-R(+8)-Pre and PH_{Crac}-mCherry, demonstrating that upon receptor activation front protein PH_{Crac} gets recruited to membrane from cytosol whereas back-associated lipid-anchored synthetic protein R(+8)-Pre maintained membrane association. Left Panels: GFP-R(+8)-Pre, right panels: PH_{Crac}-mCherry. Top left-corner: time in seconds. cAMP was added at time t=0s (also indicated by the appearance of white text “+cAMP stimulation” in the video).

File Name: Supplementary Movie 13
Description: Kinetics of cAR1 and PH_{Crac} during global receptor activation. Global cAMP stimulation driven receptor activation in *Dictyostelium* cells co-expressing cAR1-GFP and PH_{Crac}-mCherry, demonstrating that upon receptor activation front protein PH_{Crac} gets recruited to membrane from cytosol whereas uniformly distributed transmembrane protein cAR1 maintained membrane association. Left Panels: cAR1-GFP, right panels: PH_{Crac}-mCherry. Top

left-corner: time in seconds. cAMP was added at time t=0s (also indicated by the appearance of white text “+cAMP stimulation” in the video). Scale bar: 10 µm.

File Name: Supplementary Movie 14
Description: Kinetics of R(+8)-Pre and PH_{Akt} during global C5a receptor activation. Two examples of global FKP-(D-Cha)-Cha-r stimulation driven C5a receptor activation in RAW 264.7 cells co-expressing GFP-R(+8)-Pre and PH_{Akt}-mCherry, demonstrating that upon receptor activation front protein PH_{Akt} gets recruited to membrane from cytosol (and eventually the response adapts), whereas back-associated lipid-anchored protein R(+8)-Pre maintained membrane association throughout the experiment. Left Panels: GFP-R(+8)-Pre, right panels: PH_{Akt}-mCherry. Top left-corner: time in seconds. FKP-(D-Cha)-Cha-r was added at time t=0s (also indicated by the appearance of white text “+C5aR agonist” in the video). Scale bars: 10 µm.

File Name: Supplementary Movie 15
Description: Selective photoconversion of CynA suggests a shuttling mechanism for its polarized distribution. In the ventral surface of a *Dictyostelium* cell expressing CynA-KikGR, a membrane domain which is switching from back/basal to front/activated state (i.e. an area right ahead of a “shadow” wave) was photoconverted selectively using a ROI where 405 nm laser was illuminated. Note that photoconverted CynA vanished from the plane of membrane since it translocated to the cytosol, as shadow wave crossed the photoconverted area. Left Panels: CynA-KikGR (green), right panels: photoconverted CynA-KikGR (red, shown in magenta). Top left-corner: time in mm:ss format. Selective photoconversion was started at time t=0s.

File Name: Supplementary Movie 16
Description: Selective photoconversion of PTEN suggests a shuttling mechanism for its polarized distribution. In the ventral surface of a *Dictyostelium* cell expressing PTEN-KikGR, a membrane domain which is switching from back/basal to front/activated state (i.e., an area right ahead of a “shadow” wave) was photoconverted selectively using a ROI where 405 nm laser was illuminated. Note that photoconverted PTEN, vanished from the plane of membrane since it translocated to the cytosol, as shadow wave crossed the photoconverted area. Left Panels: PKBR1-KikGR (green), right panels: photoconverted PTEN-KikGR (red, shown in magenta). Top left-corner: time in mm:ss format. Selective photoconversion was started at time t=0s.

File Name: Supplementary Movie 17
Description: Selective photoconversion of PKBR1 suggests a partitioning mechanism for its polarized distribution. In the ventral surface of a *Dictyostelium* cell expressing PKBR1-KikGR, a membrane domain which is switching from back/basal to front/activated state (i.e. an area right ahead of a “shadow” wave) was photoconverted selectively using a ROI where 405 nm laser was illuminated. Note that photoconverted PKBR1, instead of disappearing from

membrane and moving to cytosol, rearranged over the plane of membrane. Left Panels: PKBR1-KikGR (green), right panels: photoconverted PKBR1-KikGR (red, shown in magenta). Top left-corner: time in mm:ss format. Selective photoconversion was started at time t=0s. Two examples are shown.

File Name: Supplementary Movie 18
Description: Selective photoconversion of Gβγ suggests a partitioning mechanism for its polarized distribution In the ventral surface of a *Dictyostelium* cell expressing KikGR-Gβγ, a membrane domain which is switching from back/basal to front/activated state (i.e. an area right ahead of a "shadow" wave) was photoconverted selectively using a ROI where 405 nm laser was illuminated. Note that photoconverted Gβγ, instead of disappearing from membrane and moving to cytosol, rearranged over the plane of membrane. Left Panels: KikGR-Gβγ (green), right panels: photoconverted KikGR-Gβγ (red, shown in magenta). Top left-corner: time in mm:ss format. Selective photoconversion was started at time t=0s. Two examples are shown.

File Name: Supplementary Movie 19
Description: Single-molecule imaging of PKBR1 and simultaneous PIP3 wave imaging. Single-molecules of PKBR1-HaloTMR (shown in green) were imaged during ventral wave propagation in a cell, which is also expressing PIP3 sensor PHD-eGFP (shown in magenta). In this multiscale imaging setup, PIP3 sensor was indicating the separate front-state (enriched in PIP3) and back-state (depleted of PIP3) regions, whereas the single-molecules of PKBR1 was recorded to compute its diffusion profiles inside front as well as back state regions. The movies are played back at the same speed as they were taken (30 frames/second).

File Name: Supplementary Movie 20
Description: Two-dimensional Stochastic simulation of an excitable network that incorporated differential diffusion dynamics of lipid-anchored proteins. The spatiotemporal patterns of F, R, B, PP, and LP demonstrate that upon firing of the excitable network, both PP and LP consistently align to asymmetric patterns. In F/B combined panel, F is in green. In all other cases, concentrations are shown in Matplotlib "Plasma" colormap. This video corresponds to Figure 6c.

File Name: Supplementary Movie 21
Description: Two-dimensional Stochastic simulation of an excitable network where differential diffusion dynamics of lipid-anchored proteins was neglected. The spatiotemporal patterns of F, R, B, PP, and LP demonstrates that upon firing of the excitable network, only PP consistently aligns to asymmetric patterns, but LP, due to its uniform diffusion along all grid points, could not undergo symmetry breaking. In F/B combined panel, F is in green. In all other cases,

concentrations were shown in Matplotlib “Plasma” colormap. This video corresponds to Supplementary Figure 13a.

File Name: Supplementary Movie 22
Description: Optogenetic recruitment of cytosolic CRY2PHR-mCherry-R+ and cytosolic CRY2PHR-mCherry(CTRL) to membrane bound cAR1-CIBN. First two movies demonstrate two examples of recruitment of cytosolic CRY2PHR-mCherry-R+ to membrane bound cAR1-CIBN demonstrating synthetically increasing affinity for the back-state region is sufficient to generate polarized pattern out of a normally uniformly distributed protein cAR1. Third movie demonstrate that CRY2PHR-mCherry(CTRL) recruitment to cAR1-CIBN does not induce any symmetry breaking. In first two movies: Left panels: CRY2PHR-mCherry-R+. In third movie: Left panels: CRY2PHR-mCherry(CTRL). In all movies: Middle panels: Lifeact-HaloTag (Janelia Flour 646); Right panels: Merged view. Top left corners showing time in second. The 488 nm laser was globally turned on at time t=0s to initiate optogenetic recruitment (also indicated by the appearance of white text “488 nm ON” in the video).

File Name: Supplementary Movie 23
Description: Optogenetic recruitment of cytosolic CRY2PHR-mCherry-R+ to membrane bound Lyn11-CIBN-GFP in HL-60 neutrophils. Optogenetic recruitment of cytosolic CRY2PHR-mCherry-R+ to membrane bound Lyn11-CIBN-GFP in a differentiated HL-60 neutrophil cell, demonstrating synthetically increasing affinity for the back-state region is sufficient to partition a uniformly distributed lipid-anchored protein Lyn11 in HL-60 cells. Left panels: Lyn11-CIBN-GFP, Middle panels: CRY2PHR-mCherry-R+, right panels: DIC images. Top left corners showing time in second. The 488 nm laser was globally turned on at the beginning of the experiment.

File Name: Supplementary Movie 24
Description: Optogenetic recruitment of cytosolic CRY2PHR-mCherry(CTRL) to membrane bound Lyn11-CIBN-GFP in HL-60 neutrophils. Optogenetic recruitment of cytosolic CRY2PHR-mCherry(CTRL) to membrane bound Lyn11-CIBN-GFP in a differentiated HL-60 neutrophil cell, demonstrating that CRY2PHR-mCherry(CTRL) recruitment does not polarize uniformly distributed lipid-anchored protein Lyn11 in HL-60 cells. Left panels: Lyn11-CIBN-GFP, Middle panels: CRY2PHR-mCherry(CTRL), right panels: DIC images. Top left corners showing time in second. The 488 nm laser was globally turned on at the beginning of the experiment.
