# Supplementary material for: A dynamic partitioning mechanism polarizes membrane protein distribution
Source: Nat Commun. 2023 Nov 30;14:7909. doi: 10.1038/s41467-023-43615-2 (PMC10689845; doi:10.1038/s41467-023-43615-2)
Supplement: Supplementary file 28 — Reporting Summary [file 41467_2023_43615_MOESM28_ESM.pdf]

nature portfolio

Corresponding author(s): Tatsat Banerjee, Peter N. Devreotes, Pablo A. Iglesias

Last updated by author(s): Nov 2, 2023

Reporting Summary

Nature Portfolio wishes to improve the reproducibility of the work that we publish. This form provides structure for consistency and transparency in reporting. For further information on Nature Portfolio policies, see our Editorial Policies and the Editorial Policy Checklist.

Statistics

For all statistical analyses, confirm that the following items are present in the figure legend, table legend, main text, or Methods section.

n/a	Confirmed	
☐	☒	The exact sample size (*n*) for each experimental group/condition, given as a discrete number and unit of measurement
☐	☒	A statement on whether measurements were taken from distinct samples or whether the same sample was measured repeatedly
☐	☒	The statistical test(s) used AND whether they are one- or two-sided<br>*Only common tests should be described solely by name; describe more complex techniques in the Methods section.*
☐	☒	A description of all covariates tested
☐	☒	A description of any assumptions or corrections, such as tests of normality and adjustment for multiple comparisons
☐	☒	A full description of the statistical parameters including central tendency (e.g. means) or other basic estimates (e.g. regression coefficient) AND variation (e.g. standard deviation) or associated estimates of uncertainty (e.g. confidence intervals)
☐	☒	For null hypothesis testing, the test statistic (e.g. *F*, *t*, *r*) with confidence intervals, effect sizes, degrees of freedom and *P* value noted<br>*Give P values as exact values whenever suitable.*
☒	☐	For Bayesian analysis, information on the choice of priors and Markov chain Monte Carlo settings
☒	☐	For hierarchical and complex designs, identification of the appropriate level for tests and full reporting of outcomes
☐	☒	Estimates of effect sizes (e.g. Cohen's *d*, Pearson's *r*), indicating how they were calculated

Our web collection on statistics for biologists contains articles on many of the points above.

Software and code

Policy information about availability of computer code

Data collection	ZEN Black (2.3 SP1 FP2); Zen Blue (2.3); NIS Elements (Ar 4.40.00 Build 1084); Andor iQ2.
Data analysis	Custom-written MATLAB codes, Fiji/ImageJ, and ilastik were used for image quantification and analysis. Custom-written MATLAB codes were developed for computational simulations. URDME framework and Xcode was used in simulations. Custom written codes are available here: https://github.com/tatsatb/Dynamic-Partitioning-of-Membrane-Proteins. MATLAB, GraphPad Prism, and OriginPro were used for statistical analysis. Following versions of software were used to perform analysis: MATLAB 2021a and MATLAB 20222a, ilastik 1.3.3post3, URDME 1.4, Xcode 12, Fiji/ImageJ 1.53q, OriginPro 9.0, Graphpad Prism 8.

For manuscripts utilizing custom algorithms or software that are central to the research but not yet described in published literature, software must be made available to editors and reviewers. We strongly encourage code deposition in a community repository (e.g. GitHub). See the Nature Portfolio guidelines for submitting code & software for further information.

Data

Policy information about availability of data

All manuscripts must include a data availability statement. This statement should provide the following information, where applicable:

- Accession codes, unique identifiers, or web links for publicly available datasets
- A description of any restrictions on data availability
- For clinical datasets or third party data, please ensure that the statement adheres to our policy

All data needed to evaluate the conclusions in the paper are present in the main text or the supplementary materials. Any additional requests for information or

data will be fulfilled by the corresponding author upon reasonable request. Source data are provided with this paper.

Human research participants

Policy information about studies involving human research participants and Sex and Gender in Research.

Reporting on sex and gender	Not Applicable.
Population characteristics	Not Applicable.
Recruitment	Not Applicable.
Ethics oversight	Not Applicable.

Note that full information on the approval of the study protocol must also be provided in the manuscript.

Field-specific reporting

Please select the one below that is the best fit for your research. If you are not sure, read the appropriate sections before making your selection.

☒ Life sciences ☐ Behavioural & social sciences ☐ Ecological, evolutionary & environmental sciences

For a reference copy of the document with all sections, see nature.com/documents/nr-reporting-summary-flat.pdf

Life sciences study design

All studies must disclose on these points even when the disclosure is negative.

Sample size	Sample sizes were chosen large enough to account for the heterogeneity among cells. Similar sample sizes were used for experiment and control group for consistency. All the experiments were performed repetitively (often with parallel imaging setup), and hence, for any plot, usually at least 200 images were available for analysis. The sample sizes that we used it this study was based on the extensive experience with similar experiments in our laboratories (Banerjee T et al. Nat Cell Biol. 2022, Miao Y et al. Mol Syst Biol. 2019, Miao Y et al. Nat Cell Biol. 2017, Pal D S et al. Dev Cell 2023, Li X et al. Proc Natl Acad Sci. U.S.A. 2018, Zhan H et al. Dev Cell 2020, Matsuoka S et al. Nat Commun. 2018, Matsuoka S et al. Biophys J. 2009, Takebayashi K et al. J Cell Sci. 2023, Vazquez F et al. Proc Natl Acad Sci. U.S.A. 2006, Ueda M et al. Science 2001, etc). Chosen sample sizes are consistent with those that were reported by other researchers in the domain as well (Riedl M et al. Nat Commun. 2023, Yang Q et al. eLife 2022, Bement W M et al. Nat Cell Biol. 2015, Flemming S et al. Proc Natl Acad Sci. U.S.A. 2020, Gerhardt M et al. J Cell Sci. 2014, Shellard A. et al. Science 2018, O'Neill P R et al. Dev Cell 2018, De Belly H et al. Cell 2023, Bisaria A et al. Science 2020, Wu Z et al. Nat Commun. 2018, Reversat A et al. Nature 2020, etc).
Data exclusions	For optogenetics experiments, for either control or experiment group, data were excluded when there is no recruitment as it essentially implies the absence of expression of the untagged membrane anchor in that particular cell. No other data were excluded from the study.
Replication	All reported findings were reliably reproduced using at least three independent biological replicates.
Randomization	This study does not involve any human or animal subjects. To generate any particular experiment or control group cell lines for subsequent imaging assays, cells were collected randomly from the parental cell lines in culture. Cells were randomized into wells for the drug treatment experiments.
Blinding	During data collection, it was not feasible, as the differences between experimental and control groups, in terms of protein expression level, localization, and dynamics, were apparent in microscopes for most subcellular symmetry breaking events. Readouts were not subjected to experimenter bias. It was not relevant during data analysis as most of the quantifications were performed by automated image analysis in MATLAB and/or Fiji/ImageJ.

Reporting for specific materials, systems and methods

We require information from authors about some types of materials, experimental systems and methods used in many studies. Here, indicate whether each material, system or method listed is relevant to your study. If you are not sure if a list item applies to your research, read the appropriate section before selecting a response.

Materials & experimental systems

n/a	Involved in the study
☐	☒ Antibodies
☐	☒ Eukaryotic cell lines
☒	☐ Palaeontology and archaeology
☒	☐ Animals and other organisms
☒	☐ Clinical data
☒	☐ Dual use research of concern

Methods

n/a	Involved in the study
☒	☐ ChIP-seq
☒	☐ Flow cytometry
☒	☐ MRI-based neuroimaging

Antibodies

Antibodies used

Anti-Bovine Serum Albumin antibody (clone BSA-33), Mouse monoclonal (Sigma-Aldrich; SAB4200688)

Validation

The antibody used is this study has been validated by manufacturer. The validation statement is available in "Product Information" document on Sigma-Aldrich website. The relevant part is quoted here:

"Anti-Bovine Serum Albumin (BSA) antibody, Mouse monoclonal (mouse IgG2a isotype) is derived from the BSA-33 hybridoma produced by the fusion of mouse myeloma cells and splenocytes from mouse BALB/c mice immunized with Bovine Serum Albumin (BSA). The isotype is determined by ELISA using Mouse Monoclonal Antibody Isotyping Reagents, Product Number ISO2. The antibody is purified from culture supernatant of hybridoma cells.

Anti-Bovine Serum Albumin (BSA) antibody, Mouse monoclonal is specific for bovine serum albumin and is highly reactive with goat and sheep serum albumins. The product is less reactive with dog, turkey and horse serum albumins. Monoclonal Anti-BSA does not cross- react with human, rabbit, guinea pig, chicken, hamster, pig, mouse, cat, pigeon, rat or donkey serum albumins. The antibody may be used in various immunochemical techniques including Immunoblotting (~70kDa), Immunoprecipitation and Indirect ELISA. Detection of the BSA band by Immunoblotting is specifically inhibited."

Eukaryotic cell lines

Policy information about cell lines and Sex and Gender in Research

Cell line source(s)

Dictyostelium AX2: Lab stock [originally gift from R.R. Kay, MRC LMB, Cambridge, UK]. Dictyostelium Gbeta—: Lab stock [origianaly generated in lab from AX2]. HL-60: Lab stock [originally gift from O.D. Weiner (University of California, San Francisco)]. RAW 264.7: Lab stock [originally gift from N. Gautam (Washington University School of Medicine, St. Louis) who obtained it from the Washington University Tissue Culture Support Center and American Type Culture Collection (ATCC, TIB-71)].

Authentication

The Orion Weiner lab, from where we obtained the HL-60 cell lines, recently authenticated these lines by STR profiling (Saha S et al. Mol. Biol. Cell 2023; De Belly H et al. Cell 2023). ATCC (the original supplier of RAW 264.7 cells) authenticate the lines using STR profiling. We confirm that all the cell lines in culture exhibited expected morphology and growth rate.

Mycoplasma contamination

No mycoplasma contamination was detected.

Commonly misidentified lines
(See ICLAC register)

No cell lines used in this study are listed in ICLAC register.
